# Dynamic response of a large-diameter end-bearing pile in permafrost

**DOI:** 10.1038/s41598-023-46639-2

**Published:** 2024-01-05

**Authors:** Qiang Li, Yongyuan Zhang, Chen Chen, Minjie Wen, Wenjie Guan, Weiwei Duan

**Affiliations:** 1 Department of Civil Engineering, Zhejiang Ocean University, Zhoushan, 316022 China; 2 School of Mechanics and Engineering Science, Shanghai University, Shanghai, 200444 China; 3 School of Civil Engineering and Architecture, Zhejiang Sci-Tech University, Hangzhou, 310018 China

**Keywords:** Civil engineering, Applied mathematics

## Abstract

Vertically dynamic model of a large-diameter pile in frozen soil is established, in which the frozen soil is described to a saturated frozen porous media, and the large diameter end-bearing pile is simplified to a one-dimensional rod considering the influence of the transverse inertia effect. Analytical solutions of the longitudinal coupling vibration between the end-bearing pile and the frozen soil are obtained using Helmholtz decomposition and variable separation methods in the frequency domain. By comparing the dynamic responses of the longitudinal vibration of the large diameter end-bearing pile with the traditionally one-dimensional pile, as well as the impedance factor of the frozen soil layer induced by the pile vibration, these demonstrate the influence of the transverse inertia effect on the high frequency vibration of large diameter pile is significant, and the influence on the pile with a smaller slenderness ratio is larger. The temperature and the Poisson’s ratio also have significant effects on the vertical vibration of large diameter piles in frozen soil, which cannot be ignored in the analysis.

## Introduction

Vertical vibration of pile due to vertically dynamic loads from earthquake, traffic and vibrating machinery is of great practical engineering significance for pile integrity test, and dynamic designs of pile in earthquake engineering, machine foundation and transportation facilities^1,2^.At present, with the increasing engineering activities in cold regions, such as highways and high-speed railways, the large diameter piles are widely used in permafrost. The current study on dynamic response of pile only investigated the vertical vibration of the pile at a normal temperature. However, the low-temperature environment will transform soil into frozen soil, resulting in drastic changes in the physical and mechanical properties of the soil, thereby affecting the dynamic interaction between the pile and soil. In order to investigate the vertically dynamic responses of pile foundation in permafrost, and evaluate the effect of environmental temperature on pile integrity test and pile driving, it is necessary to propose a vertical vibration theory suitable for large diameter piles in frozen soil. Dynamic characteristics of a large diameter pile in permafrost involve two key issues: one is the transverse inertia effect of large-diameter pile, and the other is the description of the movements of multiphase media in frozen soil.

Firstly, the traditionally vertical vibration analysis of pile is usually treated as one-dimensional Bernoulli rod (1-D rod model)^3–5^, but only for slender piles. Due to the smaller slenderness ratio of the large diameter pile, there will be a large deviation from the one-dimensional rod theory^6^. In recent years, the three-dimensional effects of piles have been confirmed by numerous theoretical studies and the field test results of piles^7,8^. By analyzing the three-dimensional effect of piles, the interaction between large diameter pile and soil is well understood through the 3-D rod theory^9–15^.However, this method is mathematically complicated in theory, and it is difficult to apply it to the pile dynamic detection. Therefore, a more practical method is required in dynamic response analysis of pile vertical vibration. A simplified model considering transverse inertial effect, which can approximately reflect the three-dimensional effect of piles without getting involved in too complex mathematical form, was proposed based on the principle of energy conservation originally proposed by Rayleigh and Love. Li et al.^16^ earlier established a dynamic interaction model between the large-diameter pile and saturated soil by taking into account the transversely inertial effect of piles. This model is simpler than the three-dimensional form and is more appropriate for the study of large diameter pile integrity detection. This model has been further developed by some researchers in recent years, and widely applied to theoretical vertical vibration analysis of large diameter pipe piles, wedge piles and defective piles^17–23^.

The second key issue is that the physical model description of permafrost involves treating the soil as a multiphase medium. In previous studies, the soil is generally regarded as a single-phase medium, neglecting the influence of the fluid in soil, and used an elastic or viscoelastic medium for dynamic interaction analysis between the pile and soil. However, it is not always accurate to analyze the soil as a single medium. Later, taking the soil around the pile as a two-phasic medium the dynamic responses of pile foundations in saturated porous medium are obtained^24–31^. Subsequently, the dynamic responses of pile in unsaturated soil was derived, in which the soil was treated as a mixture of solid, liquid and gas^32–36^ . All of the above studies, constrained soil in the normal temperature environment, however, when the temperature is lower than the freezing point, the influence of temperature on the interaction between the pile and soil cannot be ignored^37^. Leclaire et al.^38,39^ first established a saturated frozen soil model by considering the coupling between the solid, ice and liquid infrozen soil, and the validation has been verified by wave propagations in frozen porous media via experiments. This dynamic model, which is applicable to the saturated frozen soil, provides a possibility for the analysis of vertical vibration of the large diameter piles in permafrost.

In this study, based on the saturated frozen porous media theory and taking transverse inertia effect into account, a simplified coupling model of dynamic responses between the pile and soil is established and its analytical solution of a large diameter pile in permafrost is obtained. And the superiority of the model is demonstrated by comparing with the traditional 1-D rod theory. Then, the influence of the temperature and material parameters on the vertical vibration characteristics of the large-diameter pile in frozen soil is analyzed. This theoretical model is of great significance to the pile integrity detection of the large-diameter pile foundation in cold regions.

## Mathematical model

### Basic assumptions and boundary conditions

**Figure 1:**
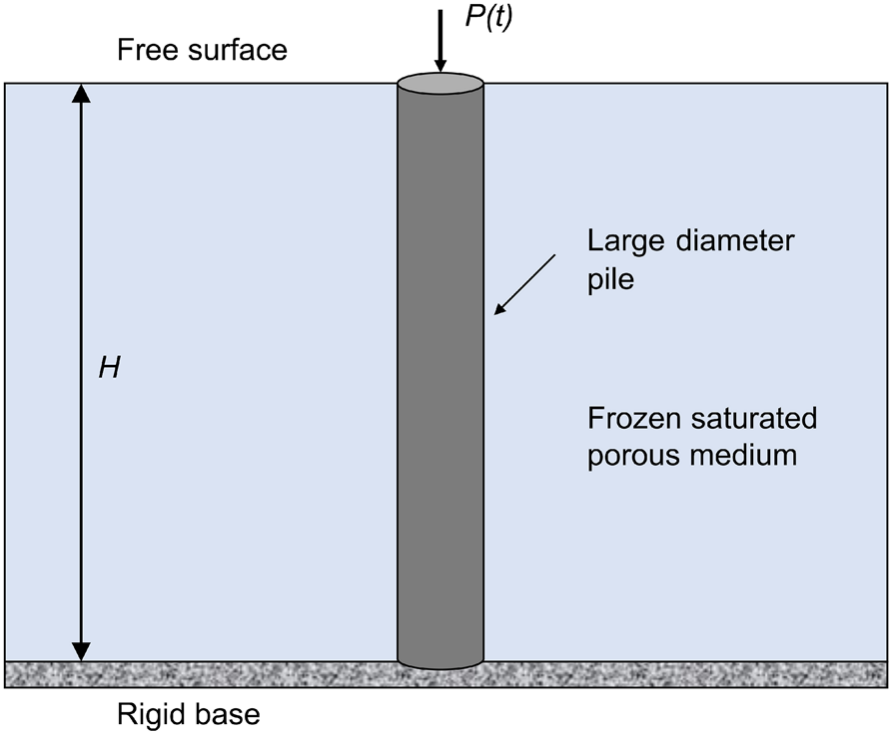
Calculation model of the large diameter pile in frozen soil.

In this section, the soil layer is simplified as a homogeneous and isotropic frozen saturated porous medium. The vertical coupling vibration of a pile in frozen saturated porous medium is investigated, with the top of pile subjected to the vertically arbitrary excitation force $P(\text {t})$. The calculation diagram of soil-pile system is shown in Fig. 1, and its basic assumptions and governing equations are described as follows:

- Based on the LCA model originated by Leclaire et al.^38^, the complex medium is composed of soil particles, ice, and liquid, in which the interactions between solid phase and ice phase are neglected. The bottom of the frozen soil layer is bedrock simplified to a rigid base, that is, the displacement of the base is zero. And the top of the frozen soil layer is a free surface, where the normal stress is equal to zero.
- The pile is an elastic and homogenous circular rod. *H* and *a* denote the length and radius of pile; ${{\rho }_{\text {p}}}$ and ${{E}_{p}}$ represent the density and elastic modulus of pile, respectively.
- The deformation of the pile is infinitesimal, and the displacements and forces of pile-soil contact surface are continuous. The frictional resistance of the soil to the pile is the resultant force of the particles and ice.

The governing equation of porous media theory is described using a displacement vector:

1 $$\begin{aligned} {\bar{R}} \nabla \nabla \cdot \vec {u}-{\bar{\mu }} \nabla \times \nabla \times \vec {u} = {\bar{\rho }} \frac{\partial ^{2} \vec {u}}{\partial t^{2}}+{\bar{A}} \frac{\partial \vec {u}}{\partial t} \end{aligned}$$

where $\vec {u}$ is the displacement field vector, and $\vec {u}={{\left\{ {{{\vec {u}}}^{(s)}}, {{{\vec {u}}}^{(f)}}, {{{\vec {u}}}^{(i)}} \right\} }^{T}}$ represents the solid, liquid and ice phase displacement of frozen saturated porous media, respectively. And the governing equations of frozen saturated porous media can also be expressed in forms of displacements of three phases as:

2 $$\begin{aligned} \left\{ \begin{matrix} \ \begin{array}{l} R_{11} \nabla \nabla \cdot \vec {u}^{(s)}-\mu _{11} \nabla \times \nabla \times \vec {u}^{(s)}+R_{12} \nabla \nabla \cdot \vec {u}^{(f)}=\rho _{11} \frac{\partial ^{2} \vec {u}^{(s)}}{\partial t^{2}}+\rho _{12} \frac{\partial ^{2} \vec {u}^{(f)}}{\partial t^{2}}+b_{11}\left( \frac{\partial \vec {u}^{(f)}}{\partial t}-\frac{\partial \vec {u}^{(s)}}{\partial t}\right) \\ \begin{array}{l} R_{12} \nabla \nabla \cdot \vec {u}^{(s)}+R_{22} \nabla \nabla \cdot \vec {u}^{(f)}+R_{23} \nabla \nabla \cdot \vec {u}^{(\textrm{i})}=\rho _{12} \frac{\partial ^{2} \vec {u}^{(s)}}{\partial t^{2}}+\rho _{22} \frac{\partial ^{2} \vec {u}^{(f)}}{\partial t^{2}}+\rho _{23} \frac{\partial ^{2} \vec {u}^{(\textrm{i})}}{\partial t^{2}}\\ -b_{11}\left( \frac{\partial \vec {u}^{(f)}}{\partial t}-\frac{\partial \vec {u}^{(s)}}{\partial t}\right) - b_{33}\left( \frac{\partial \vec {u}^{(f)}}{\partial t}-\frac{\partial \vec {u}^{(\textrm{i})}}{\partial t}\right) \end{array}\\ R_{33} \nabla \nabla \cdot \vec {u}^{({\hat{i}})}-\mu _{33} \nabla \times \nabla \times \vec {u}^{(\textrm{i})}+R_{23} \nabla \nabla \cdot \vec {u}^{(f)}=\rho _{23} \frac{\partial ^{2} \vec {u}^{(f)}}{\partial t^{2}}+\rho _{33} \frac{\partial ^{2} \vec {u}^{(\textrm{i})}}{\partial t^{2}}+b_{33}\left( \frac{\partial \vec {u}^{(f)}}{\partial t}-\frac{\partial \vec {u}^{(i)}}{\partial t}\right) \end{array} \end{matrix} \right. \ \end{aligned}$$

where

$$\begin{aligned} R_{11}= & {} K_{1}+\frac{4}{3} \mu _{11}=\left[ \left( 1-c_{1}\right) \phi _{s}\right] ^{2} K_{a v}+K_{s m}+\frac{4}{3} \mu _{11}, R_{12}=R_{21}=C_{12}=\left( 1-c_{1}\right) \phi _{s} \phi _{w} K_{a v}, R_{22}=K_{2}=\phi _{w}^{2} K_{a v},\\ R_{23}= & {} R_{32}=C_{23}=\left( 1-c_{3}\right) \phi _{i} \phi _{w} K_{a v}, R_{33}=K_{3}+\frac{4}{3} \mu _{33}=\left[ \left( 1-c_{3}\right) \phi _{i}\right] ^{2} K_{a v}+K_{i m}+\frac{4}{3} \mu _{33}, \mu _{11}=\left[ \left( 1-g_{1}\right) \phi _{s}\right] ^{2} \mu _{a v}+\mu _{s m},\\ \mu _{13}= & {} \left( 1-g_{1}\right) \left( 1-g_{3}\right) \phi _{s} \phi _{i} \mu _{a v}+{\hat{\mu }}_{s i}, \mu _{33}=\left[ \left( 1-g_{3}\right) \phi _{i}\right] ^{2} \mu _{a v}+\mu _{im}, \rho _{11}=\phi _{s} \rho _{s} a_{13}+\left( a_{12}-1\right) \phi _{w} \rho _{w}+\left( a_{31}-1\right) \phi _{i} \rho _{i},\\ \rho _{12}= & {} -\left( a_{12}-1\right) \phi _{w} \rho _{w}, \rho _{13}=-\left( a_{13}-1\right) \phi _{s} \rho _{s}-\left( a_{31}-1\right) \phi _{i} \rho _{i}, \rho _{22}=\left( a_{12}+a_{23}-1\right) \phi _{w} \rho _{w}, \rho _{23}=-\left( a_{23}-1\right) \phi _{w} \rho _{w},\\ \rho _{33}= & {} \phi _{i} \rho _{i} a_{31}+\left( a_{23}-1\right) \phi _{w} \rho _{w}+\left( a_{13}-1\right) \phi _{s} \rho _{s}, b_{11}=\eta _{D} \phi _{w}^{2} / k_{s}, b_{33}=\eta _{D} \phi _{w}^{2} / k_{i},\\ K_{a v}= & {} \left[ \left( 1-c_{1}\right) \phi _{s} / K_{s}+\phi _{w} / K_{w}+\left( 1-c_{3}\right) \phi _{i} / K_{i}\right] ^{-1}, \mu _{a v}=\left[ \left( 1-g_{1}\right) \phi _{s} / \mu _{s}+\phi _{w} / i \omega \eta _{w}+\left( 1-g_{3}\right) \phi _{i} / \mu _{i}\right] ^{-1}, c_{1}=K_{sm} / \phi _{s} K_{s},\\ c_{3}= & {} K_{i m} / \phi _{i} K_{i}, g_{1}=\mu _{s m} / \phi _{s} \mu _{s}, g_{3}=\mu _{i m} / \phi _{i} \mu _{i}, K_{\text{ im }}=K_{\text{ max } }\left[ \phi _{i} /\left( 1-\phi _{s}\right) \right] ^{3.8}, \mu _{\text{ im }}=\mu _{\max }\left[ \phi _{i} /\left( 1-\phi _{s}\right) \right] ^{3.8},\\ \mu _{s m}= & {} \left( \mu _{s m K T}-\mu _{s m 0}\right) \left[ \phi _{i} /\left( 1-\phi _{s}\right) \right] ^{3.8}+\mu _{sm0}, a_{12}=\frac{\phi _{s} \rho }{\phi _{w} \rho _{w}} r_{12}+1, a_{23}=\frac{\phi _{i} \rho ^{\prime }}{\phi _{w} \rho _{w}} r_{23}+1, a_{13}=\frac{\phi _{i} \rho ^{\prime \prime }}{\phi _{s} \rho _{s}} r_{13}+1, a_{31}=\frac{\phi _{s} \rho ^{\prime \prime }}{\phi _{i} \rho _{i}} r_{31}+1,\\ \rho= & {} \frac{\phi _{w} \rho _{w}+\phi _{i} \rho _{i}}{\phi _{w}+\phi _{i}}, \rho ^{\prime }=\frac{\phi _{w} \rho _{w}+\phi _{s} \rho _{s}}{\phi _{w}+\phi _{s}}, \rho ^{\prime \prime }=\frac{\phi _{i} \rho _{i}+\phi _{s} \rho _{s}}{\phi _{i}+\phi _{s}}, k_{s}=k_{s 0} \phi _{w}^{3} /\left( 1-\phi _{s}\right) ^{3}, k_{i}=k_{i 0}\left[ \left( 1-\phi _{s}\right) / \phi _{i}\right] ^{2}\left( \phi _{w} / \phi _{s}\right) ^{3}, \eta _{D}=\eta _{w}e^{-0.03753 T}. {{\rho }_{11}} \end{aligned}$$

is the density of soil particles, ${{\rho }_{22}}$ is the density of fluid, ${{\rho }_{33}}$ is the density of ice, ${{\rho }_{12}}$ and ${{\rho }_{13}}$ represent the interaction density of solid-liquid phase and solid-ice phase, respectively; ${{\rho }_{23}}$ is the interaction density of ice-liquid phase; ${{b}_{11}}$ and ${{b}_{33}}$ denote the viscous coupling coefficients of soil skeleton and pore fluid and of ice skeleton and pore fluid, respectively; ${{\mu }_{11}}$ and ${{\mu }_{33}}$ are the shear moduli of soil skeleton and ice, respectively; $\phi _{s}$, $\phi _{w}$ and $\phi _{i}$ represent the proportions of solid, liquid and ice phases, respectively; $K_{s}$, $K_{w}$, $K_{i}$ is the bulk moduli of solid, water, and ice,respectively; $c_{1}$ is called the bulk consolidation coefficient of solid. Further parameter descriptions can be found in references^38–42^ .

Considering the transverse inertia effect of pile, the vertical vibration governing equation of a large diameter pile can be expressed as:

3 $$\begin{aligned} {{E}_{p}}\pi {{a}^{2}}\frac{{{\partial }^{2}}{{w}_{p}}}{\partial {{z}^{2}}}-f(z)={{\rho }_{p}}\pi {{a}^{2}}({{\ddot{w}}_{p}}-{{\nu }^{2}}{{a}^{2}}\frac{{{\partial }^{2}}{{{\ddot{w}}}_{p}}}{\partial {{z}^{2}}}) \end{aligned}$$

where $f(z)=-2\pi a(\tau _{zr}^{(s)}(a,z)+\tau _{zr}^{(i)}(a,z))$, *f*(*z*) represents the friction around the pile, ${w}_{p}$ donates the displacement of the pile, $\nu$ is Poisson’s ratio of pile material.

The surface of the soil layer is a free boundary:

4 $$\begin{aligned} {{\left. {\sigma }_{z}^{(s)} \right| }_{ z=0}}=0, {{\left. {\sigma }_{z}^{(i)} \right| }_{ z=0}}=0, {{\left. {\sigma }_{z}^{(f)} \right| }_{ z=0}}=0, \end{aligned}$$

The bottom of the soil layer is the rigid supporting condition:

5 $$\begin{aligned} {{\left. {u}_{z}^{(s)} \right| }_{ z=H }}=0,{\left. {u}_{z}^{(i)} \right| }_{ z=H }=0 \end{aligned}$$

The boundary condition of soil layer on pile shaft is impervious:

6 $$\begin{aligned} \left. {u}_{r}^{(f)} \right| _{{r}=a}=0 \end{aligned}$$

Assuming that the interaction of pile and frozen soil is perfect contact, the stresses and displacements are continuous:

7 $$\begin{aligned} f(z)=-2\pi a(\tau _{zr}^{(s)}(a,z)+\tau _{zr}^{(i)}(a,z)), {u}_{z}^{(s)}(a,z)={{{w}}_{p}}(z), {u}_{z}^{(i)}(a,z)={{{w}}_{p}}(z), {u}_{r}^{(s)}(a,z)={{{w}}_{pr}}(z), {u}_{r}^{(i)}(a,z)={{{w}}_{pr}}(z) \end{aligned}$$

The boundary conditions of the pile are given as follows:

8 $$\begin{aligned} {{\left. {{{{w}}}_{p}}(z) \right| }_{ z=H }}=0,({{\left. {{E}_{p}}\frac{\partial {{w}_{p}}}{\partial z}+{{\nu }^{2}}{{\rho }_{p}}{{a}^{2}}\frac{{{\partial }^{2}}{{{\ddot{w}}}_{p}}}{\partial {{z}^{2}}}) \right| }_{z=0}}=p(t) \end{aligned}$$

### Analytical solutions of dynamic response of frozen soil.

To obtain the basic solution of dynamic response of frozen saturated porous media, the vector Helmholtz decomposition is firstly introduced into Eq. (1) :

9 $$\begin{aligned} \vec {u}=\nabla \phi +\nabla \times \vec {\psi } \end{aligned}$$

which satisfy $\nabla \cdot \vec {\psi }=0$, and where $\phi$ and $\vec {\psi }$ represent the scalar potential and vector potential of displacement, respectively. ${{\vec {\psi }}^{(k)}}={{\chi }^{(k)}}{{e}_{z}}+\nabla \times ({{\eta }^{(k)}}{{e}_{z}})$, and $\nabla \cdot ({\tilde{\eta }}{{e}_{z}})=0$, where ${{\chi }^{(k)}}$ and ${{\eta }^{(k)}}$represent two scalar potentials.

By introducing the Laplace transformation in the time domain, the Eq. (1) can be deduced as:

10 $$\begin{aligned} \left\{ \begin{matrix} [{\tilde{R}}{{\nabla }^{2}}-({\tilde{\rho }}{{s}^{2}}+{\tilde{A}}s){{a}^{2}}]{\tilde{\phi }}=0 \\ [{\tilde{\mu }}{{\nabla }^{2}}-({\tilde{\rho }}{{s}^{2}}+{\tilde{A}}s){{a}^{2}}]\tilde{\vec {\psi }}=0 \\ \end{matrix} \right. \ \end{aligned}$$

where ${\tilde{\phi }}$ and $\tilde{\vec {\psi }}$ are the scalar potential and vector potentials of displacement, respectively, and the tilde is the combination of the Laplace transform and the nondimensionalization.

Substituting Eq. (2) into (10), the governing equations in the form of potential function for frozen saturated porous media can be obtained:

11 $$\begin{aligned}{} & {} \left\{ \left[ \begin{matrix} {{\nabla }^{2}} &{} 0 &{} 0 \\ 0 &{} 0 &{} 0 \\ 0 &{} 0 &{} \mu _{33}^{*}{{\nabla }^{2}} \\ \end{matrix} \right] -\left[ \begin{matrix} {{\delta }^{2}}+b_{11}^{*}\delta &{} \rho _{12}^{*}{{\delta }^{2}}-b_{11}^{*}\delta &{} 0 \\ \rho _{12}^{*}{{\delta }^{2}}-b_{11}^{*}\delta &{} \rho _{22}^{*}{{\delta }^{2}}+(b_{11}^{*}+b_{33}^{*})\delta &{} \rho _{23}^{*}{{\delta }^{2}}-b_{33}^{*}\delta \\ 0 &{} \rho _{23}^{*}{{\delta }^{2}}-b_{33}^{*}\delta &{} \rho _{33}^{*}{{\delta }^{2}}+b_{33}^{*}\delta \\ \end{matrix} \right] \right\} \left\{ \begin{matrix} {{{\tilde{\vec {\psi }}}}^{(s)}} \\ {{{\tilde{\vec {\psi }}}}^{(f)}} \\ {{{\tilde{\vec {\psi }}}}^{(i)}} \\ \end{matrix} \right\} =0 \end{aligned}$$

12 $$\begin{aligned}{} & {} \left\{ \left[ \begin{matrix} R_{11}^{*}{{\nabla }^{2}} &{} R_{12}^{*}{{\nabla }^{2}} &{} 0 \\ R_{12}^{*}{{\nabla }^{2}} &{} R_{22}^{*}{{\nabla }^{2}} &{} R_{23}^{*}{{\nabla }^{2}} \\ 0 &{} R_{23}^{*}{{\nabla }^{2}} &{} R_{33}^{*}{{\nabla }^{2}} \\ \end{matrix} \right] -\left[ \begin{matrix} {{\delta }^{2}}+b_{11}^{*}\delta &{} \rho _{12}^{*}{{\delta }^{2}}-b_{11}^{*}\delta &{} 0 \\ \rho _{12}^{*}{{\delta }^{2}}-b_{11}^{*}\delta &{} \rho _{22}^{*}{{\delta }^{2}}+(b_{11}^{*}+b_{33}^{*})\delta &{} \rho _{23}^{*}{{\delta }^{2}}-b_{33}^{*}\delta \\ 0 &{} \rho _{23}^{*}{{\delta }^{2}}-b_{33}^{*}\delta &{} \rho _{33}^{*}{{\delta }^{2}}+b_{33}^{*}\delta \\ \end{matrix} \right] \right\} \left\{ \begin{matrix} {{\tilde{\phi }}}^{(s)} \\ {{\tilde{\phi }}}^{(f)} \\ {{\tilde{\phi }}}^{(i)} \end{matrix}\right\} =0 \end{aligned}$$

where, the dimensionless coefficients are:

$$\begin{aligned} {\tilde{R}}=\left[ \begin{matrix} R_{11}^{*} &{} R_{12}^{*} &{} 0 \\ R_{12}^{*} &{} R_{22}^{*} &{} R_{23}^{*} \\ 0 &{} R_{23}^{*} &{} R_{33}^{*} \\ \end{matrix} \right] , {\tilde{\mu }}=\left[ \begin{matrix} 1 &{} 0 &{} 0 \\ 0 &{} 0 &{} 0 \\ 0 &{} 0 &{} \mu _{33}^{*} \\ \end{matrix} \right] , {\tilde{\rho }}{{s}^{2}}{{a}^{2}}=\left[ \begin{matrix} {{\delta }^{2}} &{} \rho _{12}^{*}{{\delta }^{2}} &{} 0 \\ \rho _{12}^{*}{{\delta }^{2}} &{} \rho _{22}^{*}{{\delta }^{2}} &{} \rho _{23}^{*}{{\delta }^{2}} \\ 0 &{} \rho _{23}^{*}{{\delta }^{2}} &{} \rho _{33}^{*}{{\delta }^{2}} \\ \end{matrix} \right] , \end{aligned}$$

$$\begin{aligned} {\tilde{A}}s{{a}^{2}}= & {} \left[ \begin{matrix} b_{11}^{*}\delta &{} -b_{11}^{*}\delta &{} 0 \\ -b_{11}^{*}\delta &{} (b_{11}^{*}+b_{33}^{*})\delta &{} -b_{33}^{*}\delta \\ 0 &{} -b_{33}^{*}\delta &{} b_{33}^{*}\delta \\ \end{matrix} \right] , \\ R_{ij}^{*}= & {} \frac{{{R}_{ij}}}{{{\mu }_{11}}}, \mu _{33}^{*}=\frac{{{\mu }_{33}}}{{{\mu }_{11}}}, \delta =\sqrt{\frac{{{\rho }_{11}}}{{{\mu }_{11}}}}sa, \rho _{ij}^{*}=\frac{{{\rho }_{ij}}}{{{\rho }_{11}}}, b_{ij}^{*}=\frac{{{b}_{ij}}a}{\sqrt{{{\rho }_{11}}{{\mu }_{11}}}}, {\tilde{r}}={r}/{a}, {\tilde{z}}={z}/{a}\;, \end{aligned}$$

and *s* represents the Laplace transform parameter to time.

To obtain the nontrivial solutions, the coefficient matrices of Eqs. (11) and (12) are calculated as determinants, and the results can be expressed as:

13 $$\begin{aligned}{} & {} ({{\nabla }^{6}}+{{d}_{1}}{{\nabla }^{4}}+{{d}_{2}}{{\nabla }^{2}}+{{d}_{3}})=0 \end{aligned}$$

14 $$\begin{aligned}{} & {} ({{\nabla }^{4}}-{{d}_{4}}{{\nabla }^{2}}+{{d}_{5}})=0 \end{aligned}$$

where ${{d}_{1}}=\frac{{{a}_{1}}}{{{m}_{1}}}$, ${{d}_{2}}=\frac{{{b}_{1}}}{{{m}_{1}}}$, ${{d}_{3}}=\frac{{{c}_{1}}}{{{m}_{1}}}$, ${{d}_{4}}=\frac{-{{a}_{2}}}{{{m}_{2}}}$, ${{d}_{5}}=\frac{{{b}_{2}}}{{{m}_{2}}}$. The detailed parameter calculation results can be found in the the literature^43^. Then, the Eq. (13) can be further decomposed into Helmholtz equation, and according to the operator decomposition theory, the solution of ${{{\tilde{\phi }}}^{(k)}}$ can be obtained as follows:

15 $$\begin{aligned} {{{\tilde{\phi }}}^{(k)}}={\tilde{\phi }}_{1}^{(k)}+\tilde{\phi }_{2}^{(k)}+\tilde{\phi }_{3}^{(k)}=\sum \limits _{l=1}^{3}{{\tilde{\phi }}_{l}^{(k)}}, k=s, f, i; l=1, 2, 3 \end{aligned}$$

where ${\tilde{\phi }}_{1}^{(k)}$, ${\tilde{\phi }}_{2}^{(k)}$ and ${\tilde{\phi }}_{3}^{(k)}$ satisfy: $({{\nabla }^{2}}-\beta _{l}^{2}){\tilde{\phi }}_{l}^{(k)}=0$, $l=1, 2, 3$.

According to the separation of variables method and considering the Sommerfeld radiation conditions, it can be expressed as:

16 $$\begin{aligned} \begin{aligned} {{{{\tilde{\phi }}}}^{(k)}}&={{k}_{0}}{{g}_{11}}\tilde{r}(C_{1}^{(k)}{{e}^{{{g}_{12}}\tilde{z}}}+D_{1}^{(k)}{{e}^{-{{g}_{12}}\tilde{z}}})+{{k}_{0}}{{g}_{21}}{{\tilde{r}}}(C_{2}^{(k)}{{e}^{{{g}_{22}}\tilde{z}}}+D_{2}^{(k)}{{e}^{-{{g}_{22}}\tilde{z}}})+{{k}_{0}}{{g}_{31}}{{\tilde{r}}}(C_{3}^{(k)}{{e}^{{{g}_{32}}\tilde{z}}}+D_{3}^{(k)}{{e}^{-{{g}_{32}}{{\tilde{z}}}}}) \\&=\sum \limits _{l=1}^{3}{{{k}_{0}}{{g}_{l1}}\tilde{r}(C_{l}^{(k)}{{e}^{{{g}_{l2}}\tilde{z}}}+D_{l}^{(k)}{{e}^{-{{g}_{l2}}{{\tilde{z}}}}})} \end{aligned} \end{aligned}$$

where $g_{l1}^{2}+g_{l2}^{2}=\beta _{l}^{2}$, $l=1,2,3$; ${{K}_{0}}(g{\tilde{r}})$ represents the second kind of modified Bessel function; $C_{1}^{(k)}$, $C_{2}^{(k)}$, $C_{3}^{(k)}$,$D_{1}^{(k)}$, $D_{2}^{(k)}$ and $D_{3}^{(k)}$ denote the undetermined coefficients; $k=s,f,i$ represent the solid, liquid, and ice, respectively.

Similarly, according to the above methods, the decomposition of Eq. (14) is carried out as follows:

17 $$\begin{aligned} ({{\nabla }^{4}}-{{d}_{4}}{{\nabla }^{2}}+{{d}_{5}}){{\tilde{\vec {\psi }}}^{(k)}}=0 \rightarrow ({{\nabla }^{2}}-\beta _{4}^{2})({{\nabla }^{2}}-\beta _{5}^{2}){{\tilde{\vec {\psi }}}^{(k)}}=0 \end{aligned}$$

where $$\beta _{4,5}^{2}=\frac{{{d}_{4}}\pm \sqrt{d_{4}^{2}-4{{d}_{5}}}}{2}$$.

18 $$\begin{aligned} {{{\tilde{\chi }}}^{(k)}}= & {} {{k}_{0}}({{g}_{41}}\tilde{r})(C_{4}^{(k)}{{e}^{{{g}_{42}}\tilde{z}}}+D_{4}^{(k)}{{e}^{-{{g}_{42}}\tilde{z}}})+{{k}_{0}}({{g}_{51}}{{\tilde{r}}})(C_{5}^{(k)}{{e}^{{{g}_{52}}\tilde{z}}}+D_{5}^{(k)}{{e}^{-{{g}_{52}}{{\tilde{z}}}}}) \end{aligned}$$

19 $$\begin{aligned} {{{\tilde{\eta }}}^{(k)}}= & {} {{k}_{0}}({{g}_{41}}\tilde{r})(C_{6}^{(k)}{{e}^{{{g}_{42}}\tilde{z}}}+D_{6}^{(k)}{{e}^{-{{g}_{42}}\tilde{z}}})+{{k}_{0}}({{g}_{51}}{{\tilde{r}}})(C_{7}^{(k)}{{e}^{{{g}_{52}}\tilde{z}}}+D_{7}^{(k)}{{e}^{-{{g}_{52}}{{\tilde{z}}}}}) \end{aligned}$$

where $g_{l1}^{2}+g_{l2}^{2}=\beta _{l}^{2}$, $l=4,5$; $C_{4}^{(k)}$, $C_{5}^{(k)}$, $C_{6}^{(k)}$, $C_{7}^{(k)}$ and $D_{4}^{(k)}$, $D_{5}^{(k)}$, $D_{6}^{(k)}$, $D_{7}^{(k)}$ ($k=s,f,i$) are undetermined coefficients.

For axisymmetric problems, the displacement field of each phase of frozen saturated porous media can be obtained as:

20 $$\begin{aligned}{} & {} \begin{aligned} {\tilde{u}}_{r}^{(k)}=&-\sum \limits _{l=1}^{3}{{{g}_{l1}}{{k}_{1}}({{g}_{l1}}\tilde{r})(C_{l}^{(k)}{{e}^{{{g}_{l2}}\tilde{z}}}+D_{l}^{(k)}{{e}^{-{{g}_{l2}}{{\tilde{z}}}}})} -{{g}_{41}}{{g}_{42}}{{k}_{1}}({{g}_{41}}\tilde{r})(C_{6}^{(k)}{{e}^{{{g}_{42}}\tilde{z}}}-D_{6}^{(k)}{{e}^{-{{g}_{42}}\tilde{z}}})\\&-{{g}_{51}}{{g}_{52}}{{k}_{1}}({{g}_{51}}\tilde{r})(C_{7}^{(k)}{{e}^{{{g}_{52}}\tilde{z}}}-D_{7}^{(k)}{{e}^{-{{g}_{52}}{{\tilde{z}}}}}) \end{aligned} \end{aligned}$$

21 $$\begin{aligned}{} & {} \begin{aligned} {\tilde{u}}_{z}^{(k)}=&\sum \limits _{l=1}^{3}{{{g}_{l2}}{{k}_{0}}({{g}_{l1}}\tilde{r})(C_{l}^{(k)}{{e}^{{{g}_{l2}}\tilde{z}}}-D_{l}^{(k)}{{e}^{-{{g}_{l2}}\tilde{z}}})}-g_{41}^{2}{{k}_{0}}({{g}_{41}}\tilde{r})(C_{6}^{(k)}{{e}^{{{g}_{42}}\tilde{z}}}+D_{6}^{(k)}{{e}^{-{{g}_{42}}\tilde{z}}})\\&-g_{51}^{2}{{k}_{0}}({{g}_{51}}\tilde{r})(C_{7}^{(k)}{{e}^{{{g}_{52}}\tilde{z}}}+D_{7}^{(k)}{{e}^{-{{g}_{52}}{{\tilde{z}}}}}) \end{aligned} \end{aligned}$$

Based on the correlations in Eqs. (11) and (12), the relationships among the undetermined coefficients can be listed as follows:

22 $$\begin{aligned} \left\{ \begin{array}{ll} \frac{C_{l}^{(s)}}{C_{l}^{(f)}}={{\xi }_{l}}, \frac{D_{l}^{(s)}}{D_{l}^{(f)}}={{\xi }_{l}} \\ \frac{C_{l}^{(i)}}{C_{l}^{(f)}}={{{{\xi }'}}_{l}},\frac{D_{l}^{(i)}}{D_{l}^{(f)}}={{{{\xi }'}}_{l}}\\ \end{array} \right. , l=1,2,3 \end{aligned}$$

where $${{\xi }_{l}}=-\frac{[R_{12}^{*}\beta _{l}^{2}-(\rho _{12}^{*}{{\delta }^{2}}b_{11}^{*}\delta )]}{[R_{11}^{*}\beta _{l}^{2}-({{\delta }^{2}}+b_{11}^{*}\delta )]}$$, $${{{\xi }'}_{l}}=-\frac{[R_{23}^{*}\beta _{l}^{2}-(\rho _{23}^{*}{{\delta }^{2}}-b_{33}^{*}\delta )]}{[R_{33}^{*}\beta _{l}^{2}-(\rho _{33}^{*}{{\delta }^{2}}+b_{33}^{*}\delta )]}$$.

23 $$\begin{aligned} \left\{ \begin{array}{ll} \frac{C_{m}^{(s)}}{C_{m}^{(f)}}={{\xi }_{m}}, \frac{D_{m}^{(s)}}{D_{m}^{(f)}}={{\xi }_{m}} \\ \frac{C_{m}^{(i)}}{C_{m}^{(f)}}={{{{\xi }'}}_{l}},\frac{D_{m}^{(i)}}{D_{m}^{(f)}}={{{{\xi }'}}_{m}}\\ \end{array} \right. ,m=4,5,6,7 \end{aligned}$$

where $${{\zeta }_{m}}=\frac{\rho _{12}^{*}{{\delta }^{2}}-b_{11}^{*}\delta }{[\beta _{m}^{2}-({{\delta }^{2}}+b_{11}^{*}\delta )]}$$, $${{{\zeta }'}_{m}}=\frac{\rho _{23}^{*}{{\delta }^{2}}-b_{33}^{*}\delta }{[\mu _{33}^{*}\beta _{m}^{2}-(\rho _{33}^{*}{{\delta }^{2}}+b_{33}^{*}\delta )]}$$.

For simplicity, the expressions for the displacement and stress fields can be found in Supplementary Equations, where the undetermined coefficients should be determined by the boundary conditions and the pile-soil coupling conditions.

By using the free boundary conditions and noting the relationships given in Eqs. (22) and (23), it can be derived as follows:

24 $$\begin{aligned} \left\{ \begin{array}{ll} (C_{1}^{(f)}+D_{1}^{(f)})=0;(C_{2}^{(f)}+D_{2}^{(f)})=0;(C_{3}^{(f)}+D_{3}^{(f)})=0; \\ (C_{6}^{(f)}-D_{6}^{(f)})=0;(C_{7}^{(f)}-D_{7}^{(f)})=0;\\ \end{array} \right. \end{aligned}$$

The expression of the vertical displacement is substituted into the formula of rigid support condition at the bottom, and then:

25 $$\begin{aligned} {{h}_{n}}=\frac{2n-1}{2\theta }\pi i, ({{h}_{n}}={{g}_{12}}={{g}_{22}}={{g}_{32}}={{g}_{42}}={{g}_{52}}) \end{aligned}$$

where $$\theta =H/a$$.

After substituting the expression for radial displacement into the equation of pile side displacement boundary conditions, therefore, the shear stresses, the vertical displacements and the radial displacements of pile-soil contact surface are expressed as follows:

26 $$\begin{aligned} {{\left. {\tilde{\tau }}_{zr}^{(s)} \right| }_{{\tilde{r}}=1}}=\sum \limits _{n=1}^{\infty }{({{\eta }_{1n}}C_{1}^{(f)}+{{\eta }_{2n}}C_{3}^{(f)}+{{\eta }_{3n}}C_{6}^{(f)}+{{\eta }_{4n}}C_{7}^{(f)})\cosh ({{h}_{n}}\tilde{z})} \end{aligned}$$

where

$$\begin{aligned} {{\eta }_{1n}}= & {} 4{{g}_{11}}{{h}_{n}}({{\xi }_{2}}-{{\xi }_{1}}){{k}_{1}}({{g}_{11}}), {{\eta }_{2n}}=4{{g}_{31}}{{h}_{n}}({{\xi }_{2}}-{{\xi }_{3}}){{k}_{1}}({{g}_{31}}),\\ {{\eta }_{3n}}= & {} [2{{g}_{41}}h_{n}^{2}(2{{\xi }_{2}}-{{\zeta }_{6}})+2g_{41}^{3}{{\zeta }_{6}}]{{k}_{1}}({{g}_{41}}), {{\eta }_{4n}}=[2{{g}_{51}}h_{n}^{2}(2{{\xi }_{2}}-{{\zeta }_{7}})+2g_{51}^{3}{{\zeta }_{7}}]{{k}_{1}}({{g}_{51}}). \end{aligned}$$

27 $$\begin{aligned} {{\left. {\tilde{\tau }}_{zr}^{(i)} \right| }_{{\tilde{r}}=1}}=\sum \limits _{n=1}^{\infty }{({{\eta }_{5n}}C_{1}^{(f)}+{{\eta }_{6n}}C_{3}^{(f)}+{{\eta }_{7n}}C_{6}^{(f)}+{{\eta }_{8n}}C_{7}^{(f)})\cosh ({{h}_{n}}\tilde{z})} \end{aligned}$$

where

$$\begin{aligned} {{\eta }_{5n}}= & {} 4\mu _{3}^{*}{{g}_{11}}{{h}_{n}}({{{\xi }'}_{2}}-{{{\xi }'}_{1}}){{k}_{1}}({{g}_{11}}), {{\eta }_{6n}}=4\mu _{3}^{*}{{g}_{31}}{{h}_{n}}({{{\xi }'}_{2}}-{{{\xi }'}_{3}}){{k}_{1}}({{g}_{31}}),\\ {{\eta }_{7n}}= & {} [2\mu _{3}^{*}{{g}_{41}}h_{n}^{2}(2{{{\xi }'}_{2}}-{{{\zeta }'}_{6}})+2\mu _{3}^{*}g_{41}^{3}{{{\zeta }'}_{6}}]{{k}_{1}}({{g}_{41}}), {{\eta }_{8n}}=[2\mu _{3}^{*}{{g}_{51}}h_{n}^{2}(2{{{\xi }'}_{2}}-{{{\zeta }'}_{7}})+2\mu _{3}^{*}g_{51}^{3}{{{\zeta }'}_{7}}]{{k}_{1}}({{g}_{51}}). \end{aligned}$$

28 $$\begin{aligned} {{\left. {\tilde{u}}_{z}^{(s)} \right| }_{{\tilde{r}}=1}}=\sum \limits _{n=1}^{\infty }{({{\eta }_{9n}}C_{1}^{(f)}+{{\eta }_{10n}}C_{3}^{(f)}+{{\eta }_{11n}}C_{6}^{(f)}+{{\eta }_{12n}}C_{7}^{(f)})\cosh ({{h}_{n}}{{\tilde{z}}})} \end{aligned}$$

where

$$\begin{aligned} {{\eta }_{9n}}= & {} 2\frac{{{h}_{n}}{{g}_{21}}{{\xi }_{1}}{{k}_{0}}({{g}_{11}}){{k}_{1}}({{g}_{21}})-{{h}_{n}}{{g}_{11}}{{\xi }_{2}}{{k}_{0}}({{g}_{21}}){{k}_{1}}({{g}_{11}})}{{{g}_{21}}{{k}_{1}}({{g}_{21}})}, {{\eta }_{10n}}=2\frac{{{h}_{n}}{{g}_{21}}{{\xi }_{3}}{{k}_{0}}({{g}_{31}}){{k}_{1}}({{g}_{21}})-{{h}_{n}}{{g}_{31}}{{\xi }_{2}}{{k}_{0}}({{g}_{21}}){{k}_{1}}({{g}_{31}})}{{{g}_{21}}{{k}_{1}}({{g}_{21}})},\\ {{\eta }_{11n}}= & {} -2\frac{{{g}_{41}}h_{n}^{2}{{\xi }_{2}}{{k}_{0}}({{g}_{21}}){{k}_{1}}({{g}_{41}})+{{g}_{21}}g_{41}^{2}{{\zeta }_{6}}{{k}_{0}}({{g}_{41}}){{k}_{1}}({{g}_{21}})}{{{g}_{21}}{{k}_{1}}({{g}_{21}})}, {{\eta }_{12n}}=-2\frac{{{g}_{51}}h_{n}^{2}{{\xi }_{2}}{{k}_{0}}({{g}_{21}}){{k}_{1}}({{g}_{51}})+{{g}_{21}}g_{51}^{2}{{\zeta }_{7}}{{k}_{0}}({{g}_{51}}){{k}_{1}}({{g}_{21}})}{{{g}_{21}}{{k}_{1}}({{g}_{21}})}. \end{aligned}$$

29 $$\begin{aligned} {{\left. {\tilde{u}}_{z}^{(i)} \right| }_{{\tilde{r}}=1}}=\sum \limits _{n=1}^{\infty }{({{\eta }_{13n}}C_{1}^{(f)}+{{\eta }_{1n}}C_{3}^{(f)}+{{\eta }_{15n}}C_{6}^{(f)}+{{\eta }_{16n}}C_{7}^{(f)})\cosh ({{h}_{n}}{{\tilde{z}}})} \end{aligned}$$

where

$$\begin{aligned} {{\eta }_{13n}}= & {} 2\frac{{{h}_{n}}{{g}_{21}}{{{{\xi }'}}_{1}}{{k}_{0}}({{g}_{11}}){{k}_{1}}({{g}_{21}})-{{h}_{n}}{{g}_{11}}{{{{\xi }'}}_{2}}{{k}_{0}}({{g}_{21}}){{k}_{1}}({{g}_{11}})}{{{g}_{21}}{{k}_{1}}({{g}_{21}})}, {{\eta }_{14n}}=2\frac{{{h}_{n}}{{g}_{21}}{{{{\xi }'}}_{3}}{{k}_{0}}({{g}_{31}}){{k}_{1}}({{g}_{21}})-{{h}_{n}}{{g}_{31}}{{{{\xi }'}}_{2}}{{k}_{0}}({{g}_{21}}){{k}_{1}}({{g}_{31}})}{{{g}_{21}}{{k}_{1}}({{g}_{21}})},\\ {{\eta }_{15n}}= & {} -2\frac{{{g}_{41}}h_{n}^{2}{{{{\xi }'}}_{2}}{{k}_{0}}({{g}_{21}}){{k}_{1}}({{g}_{41}})+{{g}_{21}}g_{41}^{2}{{{{\zeta }'}}_{6}}{{k}_{0}}({{g}_{41}}){{k}_{1}}({{g}_{21}})}{{{g}_{21}}{{k}_{1}}({{g}_{21}})}, {{\eta }_{16n}}=-2\frac{{{g}_{51}}h_{n}^{2}{{{{\xi }'}}_{2}}{{k}_{0}}({{g}_{21}}){{k}_{1}}({{g}_{51}})+{{g}_{21}}g_{51}^{2}{{{{\zeta }'}}_{7}}{{k}_{0}}({{g}_{51}}){{k}_{1}}({{g}_{21}})}{{{g}_{21}}{{k}_{1}}({{g}_{21}})}. \end{aligned}$$

The radial displacements of pile-soil contact surface can be obtained as follows:

30 $$\begin{aligned} {{\left. {\tilde{u}}_{r}^{(s)} \right| }_{{\tilde{r}}=1}}=\sum \limits _{n=1}^{\infty }{({{\eta }_{17n}}C_{1}^{(f)}+{{\eta }_{18n}}C_{3}^{(f)}+{{\eta }_{19n}}C_{6}^{(f)}+{{\eta }_{20n}}C_{7}^{(f)})\sinh ({{h}_{n}}{{\tilde{z}}})} \end{aligned}$$

where

$$\begin{aligned} {{\eta }_{17n}}=2{{g}_{11}}({{\xi }_{2}}-{{\xi }_{1}}){{k}_{1}}({{g}_{11}}), {{\eta }_{18n}}=2{{g}_{31}}({{\xi }_{2}}-{{\xi }_{3}}){{k}_{1}}({{g}_{31}}),\\ {{\eta }_{19n}}= & {} 2{{g}_{41}}{{h}_{n}}({{\xi }_{2}}-{{\zeta }_{6}}){{k}_{1}}({{g}_{41}}), {{\eta }_{20n}}=2{{g}_{51}}{{h}_{n}}({{\xi }_{2}}-{{\zeta }_{7}}){{k}_{1}}({{g}_{51}}) \end{aligned}$$

31 $$\begin{aligned} {{\left. {\tilde{u}}_{r}^{(i)} \right| }_{{\tilde{r}}=1}}=\sum \limits _{n=1}^{\infty }{({{\eta }_{21n}}C_{1}^{(f)}+{{\eta }_{22n}}C_{3}^{(f)}+{{\eta }_{23n}}C_{6}^{(f)}+{{\eta }_{24n}}C_{7}^{(f)})\sinh ({{h}_{n}}{{\tilde{z}}})} \end{aligned}$$

where

$$\begin{aligned} {{\eta }_{21n}}= & {} 2{{g}_{11}}({{{\xi }'}_{2}}-{{{\xi }'}_{1}}){{k}_{1}}({{g}_{11}}), {{\eta }_{22n}}=2{{g}_{31}}({{{\xi }'}_{2}}-{{{\xi }'}_{3}}){{k}_{1}}({{g}_{31}}),\\ {{\eta }_{23n}}= & {} 2{{g}_{41}}{{h}_{n}}({{{\xi }'}_{2}}-{{{\zeta }'}_{6}}){{k}_{1}}({{g}_{41}}), {{\eta }_{24n}}=2{{g}_{51}}{{h}_{n}}({{{\xi }'}_{2}}-{{{\zeta }'}_{7}}){{k}_{1}}({{g}_{51}}). \end{aligned}$$

However, there are still four undetermined coefficients in the above categories: $C_{1}^{(f)}$, $C_{3}^{(f)}$, $C_{6}^{(f)}$ and $C_{7}^{(f)}$, which should be further determined by the pile-soil contact conditions.

### Solution to the governing equation of pile

By using Laplace transformation and nondimensionalization, the vertical vibration equation of pile will be changed as:

32 $$\begin{aligned} \frac{{{d}^{2}}{{{{\tilde{w}}}}_{p}}}{d{{{{\tilde{z}}}}^{2}}}-\frac{\rho _{b}^{*}{{\delta }^{2}}}{E_{p}^{*}+\rho _{p}^{*}{{\delta }^{2}}{{\nu }^{2}}}{{{\tilde{w}}}_{p}}+\frac{2}{E_{p}^{*}+\rho _{p}^{*}{{\delta }^{2}}{{\nu }^{2}}}({{\left. {\tilde{\tau }}_{zr}^{(s)} \right| }_{{\tilde{r}}=1}}+{{\left. {\tilde{\tau }}_{zr}^{(i)} \right| }_{{\tilde{r}}=1}})=0 \end{aligned}$$

where $$\rho _{p}^{*}={{{\rho }_{p}}}/{{{\rho }_{11}}}\;$$, $$E_{p}^{*}={{{E}_{p}}}/{{{\mu }_{11}}}\;$$, $$\begin{aligned} {{\left. {\tilde{\tau }}_{zr}^{(s)} \right| }_{{\tilde{r}}=1}}+{{\left. {\tilde{\tau }}_{zr}^{(i)} \right| }_{{\tilde{r}}=1}}=\sum \limits _{n=1}^{\infty }{[({{\eta }_{1n}}+{{\eta }_{5n}})C_{1}^{(f)}+({{\eta }_{2n}}+{{\eta }_{6n}})C_{3}^{(f)}+({{\eta }_{3n}}+{{\eta }_{7n}})C_{6}^{(f)}+({{\eta }_{4n}}+{{\eta }_{8n}})C_{7}^{(f)}]\cosh ({{h}_{n}}{{\tilde{z}}})}. \end{aligned}$$

Because the pile satisfies the boundary conditions (initial static conditions have been used in Laplace transformation), which can be expressed as follows by Laplace transformation and nondimensionalization:

33 $$\begin{aligned} {{\left. {{{{\tilde{w}}}}_{p}} \right| }_{{{\tilde{z}}}=\theta }}=0, ({{\left. E_{p}^{*}+\rho _{p}^{*}{{\delta }^{2}}{{\nu }^{2}})\frac{\partial {{{{\tilde{w}}}}_{p}}}{\partial {\tilde{z}}} \right| }_{{{\tilde{z}}}=0}}=\frac{{\tilde{p}}(s)}{{{\mu }_{11}}} \end{aligned}$$

Equation (32) is a nonhomogeneous second-order ordinary differential equation, the solution of which can be obtained as follows:

34 $$\begin{aligned} {{{\tilde{w}}}_{p}}(z)={{A}_{1}}{{e}^{\kappa {\tilde{z}}}}+{{B}_{1}}{{e}^{-\kappa {\tilde{z}}}}+\sum \limits _{n=1}^{\infty }{\frac{-2[({{\eta }_{1n}}+{{\eta }_{5n}})C_{1}^{(f)}+({{\eta }_{2n}}+{{\eta }_{6n}})C_{3}^{(f)}+({{\eta }_{3n}}+{{\eta }_{7n}})C_{6}^{(f)}+({{\eta }_{4n}}+{{\eta }_{8n}})C_{7}^{(f)}]}{(E_{p}^{*}+\rho _{p}^{*}{{\delta }^{2}}{{\nu }^{2}})(h_{n}^{2}-{{\kappa }^{2}})}}\cosh ({{h}_{n}}{{\tilde{z}}}) \end{aligned}$$

where $\kappa =\sqrt{{\rho _{p}^{*}}/{(E_{p}^{*}+\rho _{p}^{*}{{\delta }^{2}}{{\nu }^{2}})}\;}\delta$, ${{A}_{1}}$ and ${{B}_{1}}$ donate the undetermined coefficients.

Using the pile-soil contact condition and the boundary condition at the pile top, the vertical and radial displacements of the pile can be expressed as:

35 $$\begin{aligned}{} & {} \begin{aligned} {{{{\tilde{w}}}}_{p}}=&\frac{{\tilde{P}}(s){{e}^{\kappa {\tilde{z}}}}}{{{\mu }_{11}}\kappa (E_{p}^{*} +\rho _{b}^{*}{{\delta }^{2}}{{\nu }^{2}})(1+{{e}^{2\kappa \theta }})} -\frac{{\tilde{P}}(s){{e}^{-\kappa {\tilde{z}}}}}{{{\mu }_{11}}\kappa (E_{p}^{*} +\rho _{p}^{*}{{\delta }^{2}}{{\nu }^{2}})(1+{{e}^{-2\kappa \theta }})} \\&+\sum \limits _{n=1}^{\infty }{\frac{-2[({{\eta }_{1n}}+{{\eta }_{5n}})C_{1}^{(f)} +({{\eta }_{2n}}+{{\eta }_{6n}})C_{3}^{(f)}+({{\eta }_{3n}}+{{\eta }_{7n}})C_{6}^{(f)} +({{\eta }_{4n}}+{{\eta }_{8n}})C_{7}^{(f)}]}{(E_{p}^{*}+\rho _{p}^{*}{{\delta }^{2}}{{\nu }^{2}}) (h_{n}^{2}-{{\kappa }^{2}})}}\cosh ({{h}_{n}}{{\tilde{z}}}) \end{aligned} \end{aligned}$$

36 $$\begin{aligned}{} & {} \begin{aligned} {{\left. {{{{\tilde{w}}}}_{pr}} \right| }_{{{\tilde{r}}}=1}}=&-\nu [\frac{{\tilde{p}}{{e}^{\kappa {{\tilde{z}}}}}}{\mu ({{E}_{p}}+\rho _{p}^{*}{{\delta }^{2}}{{\nu }^{2}})(1+{{e}^{2\kappa \theta }})}+\frac{{\tilde{p}}{{e}^{-\kappa {{\tilde{z}}}}}}{\mu ({{E}_{p}}+\rho _{p}^{*}{{\delta }^{2}}{{\nu }^{2}})(1+{{e}^{-2\kappa \theta }})} \\&+\sum \limits _{n=1}^{\infty }{\frac{-2{{h}_{n}}[({{\eta }_{1n}}+{{\eta }_{5n}})C_{1}^{(f)}+({{\eta }_{2n}}+{{\eta }_{6n}})C_{3}^{(f)}+({{\eta }_{3n}}+{{\eta }_{7n}})C_{6}^{(f)}+({{\eta }_{4n}}+{{\eta }_{8n}})C_{7}^{(f)}]}{({{E}_{p}}+\rho _{p}^{*}{{\delta }^{2}}{{\nu }^{2}})(h_{n}^{2}-{{\kappa }^{2}})}\sinh ({{h}_{n}}{{\tilde{z}}})}] \\ \end{aligned} \end{aligned}$$

According to the conditions of perfect contact between pile and soil and by considering the orthogonality of functions $\cosh ({{h}_{n}}{{\tilde{z}}})$ and $\sinh ({{h}_{n}}{{\tilde{z}}})$, a set of algebraic equations with four coefficients, which can determine as:

37 $$\begin{aligned}{} & {} ({{{\tilde{\mu }}}_{1n}}C_{1n}^{(f)}+{{\tilde{\mu }}_{2n}}C_{3n}^{(f)}+{{{\tilde{\mu }}}_{3n}}C_{6n}^{(f)}+{{\tilde{\mu }}_{4n}}C_{7n}^{(f)})=\frac{2}{\theta }\frac{{\tilde{P}}(s)}{{{\mu }_{11}}(E_{b}^{*}+\rho _{p}^{*}{{\delta }^{2}}{{\nu }^{2}})(h_{n}^{2}-{{\kappa }^{2}})} \end{aligned}$$

38 $$\begin{aligned}{} & {} ({{{\tilde{\mu }}}_{5n}}C_{1n}^{(f)}+{{\tilde{\mu }}_{6n}}C_{3n}^{(f)}+{{{\tilde{\mu }}}_{7n}}C_{6n}^{(f)}+{{\tilde{\mu }}_{8n}}C_{7n}^{(f)})=\frac{2}{\theta }\frac{{\tilde{P}}(s)}{{{\mu }_{11}}(E_{p}^{*}+\rho _{p}^{*}{{\delta }^{2}}{{\nu }^{2}})(h_{n}^{2}-{{\kappa }^{2}})} \end{aligned}$$

39 $$\begin{aligned}{} & {} ({{{\tilde{\mu }}}_{9n}}C_{1n}^{(f)}+{{\tilde{\mu }}_{10n}}C_{3n}^{(f)}+{{\tilde{\mu }}_{11n}}C_{6n}^{(f)}+{{\tilde{\mu }}_{12n}}C_{7n}^{(f)})=-\frac{2}{\theta }\frac{\nu {{h}_{n}}{\tilde{P}}(s)}{{{\mu }_{11}}(E_{p}^{*}+\rho _{p}^{*}{{\delta }^{2}}{{\nu }^{2}})(h_{n}^{2}-{{\kappa }^{2}})} \end{aligned}$$

40 $$\begin{aligned}{} & {} ({{{\tilde{\mu }}}_{13n}}C_{1n}^{(f)}+{{\tilde{\mu }}_{14n}}C_{3n}^{(f)}+{{\tilde{\mu }}_{15n}}C_{6n}^{(f)}+{{\tilde{\mu }}_{16n}}C_{7n}^{(f)})=-\frac{2}{\theta }\frac{\nu {{h}_{n}}{\tilde{P}}(s)}{{{\mu }_{11}}(E_{p}^{*}+\rho _{p}^{*}{{\delta }^{2}}{{\nu }^{2}})(h_{n}^{2}-{{\kappa }^{2}})} \end{aligned}$$

where

$$\begin{aligned} {{{\tilde{\mu }}}_{1n}}={{\eta }_{9n}}+\frac{2({{\eta }_{1n}}+{{\eta }_{5n}})}{(E_{p}^{*}+\rho _{p}^{*}{{\delta }^{2}}{{\nu }^{2}})(h_{n}^{2}-{{\kappa }^{2}})}, {{{\tilde{\mu }}}_{2n}}={{\eta }_{10n}}+\frac{2({{\eta }_{2n}}+{{\eta }_{6n}})}{(E_{p}^{*}+\rho _{p}^{*}{{\delta }^{2}}{{\nu }^{2}})(h_{n}^{2}-{{\kappa }^{2}})},\\ {{{\tilde{\mu }}}_{3n}}={{\eta }_{11n}}+\frac{2({{\eta }_{3n}}+{{\eta }_{7n}})}{(E_{b}^{*}+\rho _{b}^{*}{{\delta }^{2}}{{\nu }^{2}})(h_{n}^{2}-{{\kappa }^{2}})}, {{{\tilde{\mu }}}_{4n}}={{\eta }_{12n}}+\frac{2({{\eta }_{4n}}+{{\eta }_{8n}})}{(E_{p}^{*}+\rho _{p}^{*}{{\delta }^{2}}{{\nu }^{2}})(h_{n}^{2}-{{\kappa }^{2}})},\\ {{{\tilde{\mu }}}_{5n}}={{\eta }_{13n}}+\frac{2({{\eta }_{1n}}+{{\eta }_{5n}})}{(E_{p}^{*}+\rho _{p}^{*}{{\delta }^{2}}{{\nu }^{2}})(h_{n}^{2}-{{\kappa }^{2}})}, {{{\tilde{\mu }}}_{6n}}={{\eta }_{14n}}+\frac{2({{\eta }_{2n}}+{{\eta }_{6n}})}{(E_{p}^{*}+\rho _{p}^{*}{{\delta }^{2}}{{\nu }^{2}})(h_{n}^{2}-{{\kappa }^{2}})},\\ {{{\tilde{\mu }}}_{7n}}={{\eta }_{15n}}+\frac{2({{\eta }_{3n}}+{{\eta }_{7n}})}{(E_{p}^{*}+\rho _{b}^{*}{{\delta }^{2}}{{\nu }^{2}})(h_{n}^{2}-{{\kappa }^{2}})}, {{{\tilde{\mu }}}_{8n}}={{\eta }_{16n}}+\frac{2({{\eta }_{4n}}+{{\eta }_{8n}})}{(E_{p}^{*}+\rho _{p}^{*}{{\delta }^{2}}{{\nu }^{2}})(h_{n}^{2}-{{\kappa }^{2}})},\\ {{{\tilde{\mu }}}_{9n}}={{\eta }_{17n}}-\frac{2\nu {{h}_{n}}({{\eta }_{1n}}+{{\eta }_{5n}})}{(E_{p}^{*}+\rho _{p}^{*}{{\delta }^{2}}{{\nu }^{2}})(h_{n}^{2}-{{\kappa }^{2}})}, {{\tilde{\mu }}_{10n}}={{\eta }_{18n}}-\frac{2\nu {{h}_{n}}({{\eta }_{2n}}+{{\eta }_{6n}})}{(E_{p}^{*}+\rho _{p}^{*}{{\delta }^{2}}{{\nu }^{2}})(h_{n}^{2}-{{\kappa }^{2}})},\\ {{{\tilde{\mu }}}_{11n}}={{\eta }_{19n}}-\frac{2\nu {{h}_{n}}({{\eta }_{3n}}+{{\eta }_{7n}})}{(E_{p}^{*}+\rho _{p}^{*}{{\delta }^{2}}{{\nu }^{2}})(h_{n}^{2}-{{\kappa }^{2}})}, {{\tilde{\mu }}_{12n}}={{\eta }_{20n}}-\frac{2\nu {{h}_{n}}({{\eta }_{4n}}+{{\eta }_{8n}})}{(E_{p}^{*}+\rho _{p}^{*}{{\delta }^{2}}{{\nu }^{2}})(h_{n}^{2}-{{\kappa }^{2}})},\\ {{{\tilde{\mu }}}_{13n}}={{\eta }_{21n}}-\frac{2\nu {{h}_{n}}({{\eta }_{1n}}+{{\eta }_{5n}})}{(E_{p}^{*}+\rho _{p}^{*}{{\delta }^{2}}{{\nu }^{2}})(h_{n}^{2}-{{\kappa }^{2}})}, {{\tilde{\mu }}_{14n}}={{\eta }_{22n}}-\frac{2\nu {{h}_{n}}({{\eta }_{2n}}+{{\eta }_{6n}})}{(E_{p}^{*}+\rho _{p}^{*}{{\delta }^{2}}{{\nu }^{2}})(h_{n}^{2}-{{\kappa }^{2}})},\\ {{{\tilde{\mu }}}_{15n}}={{\eta }_{23n}}-\frac{2\nu {{h}_{n}}({{\eta }_{3n}}+{{\eta }_{7n}})}{(E_{p}^{*}+\rho _{p}^{*}{{\delta }^{2}}{{\nu }^{2}})(h_{n}^{2}-{{\kappa }^{2}})}, {{\tilde{\mu }}_{16n}}={{\eta }_{24n}}-\frac{2\nu {{h}_{n}}({{\eta }_{4n}}+{{\eta }_{8n}})}{(E_{p}^{*}+\rho _{p}^{*}{{\delta }^{2}}{{\nu }^{2}})(h_{n}^{2}-{{\kappa }^{2}})}. \end{aligned}$$

Assuming that ${{{\tilde{p}}}^{*}}(s)=\frac{{\tilde{p}}(s)}{{{\mu }_{11}}}$, and then:

41 $$\begin{aligned}{} & {} \begin{aligned} {{{{\tilde{w}}}}_{p}}=&\frac{{{{{\tilde{P}}}}^{*}}(s){{e}^{\kappa {\tilde{z}}}}}{\kappa (E_{p}^{*}+\rho _{p}^{*}{{\delta }^{2}}{{\nu }^{2}})(1+{{e}^{2\kappa \theta }})}-\frac{{{{{\tilde{P}}}}^{*}}(s){{e}^{-\kappa {\tilde{z}}}}}{\kappa (E_{p}^{*}+\rho _{p}^{*}{{\delta }^{2}}{{\nu }^{2}})(1+{{e}^{-2\kappa \theta }})} \\&+\sum \limits _{n=1}^{\infty }{\frac{-2[({{\eta }_{1n}}+{{\eta }_{5n}})C_{1}^{(f)}+({{\eta }_{2n}}+{{\eta }_{6n}})C_{3}^{(f)}+({{\eta }_{3n}}+{{\eta }_{7n}})C_{6}^{(f)}+({{\eta }_{4n}}+{{\eta }_{8n}})C_{7}^{(f)}]}{(E_{p}^{*}+\rho _{p}^{*}{{\delta }^{2}}{{\nu }^{2}})(h_{n}^{2}-{{\kappa }^{2}})}}\cosh ({{h}_{n}}{{\tilde{z}}}) \\ \end{aligned} \end{aligned}$$

42 $$\begin{aligned}{} & {} \begin{aligned} {\tilde{P}}(z)=&({{E}_{p}}+\rho _{p}^{*}{{\delta }^{2}}{{\nu }^{2}})\frac{\partial {{{{\tilde{w}}}}_{p}}}{\partial {\tilde{z}}}=\frac{{{{{\tilde{P}}}}^{*}}{{e}^{\kappa {\tilde{z}}}}}{1+{{e}^{2\kappa \theta }}}+\frac{{{{{\tilde{P}}}}^{*}}{{e}^{-\kappa {\tilde{z}}}}}{1+{{e}^{-2\kappa \theta }}} \\&+ \sum \limits _{n=1}^{\infty }{\frac{-2{{h}_{n}}[({{\eta }_{1n}}+{{\eta }_{5n}})C_{1}^{(f)}+({{\eta }_{2n}}+{{\eta }_{6n}})C_{3}^{(f)}+({{\eta }_{3n}}+{{\eta }_{7n}})C_{6}^{(f)}+({{\eta }_{4n}}+{{\eta }_{8n}})C_{7}^{(f)}]}{(h_{n}^{2}-{{\kappa }^{2}})}}\sinh ({{h}_{n}}{{\tilde{z}}}) \\ \end{aligned} \end{aligned}$$

The displacement admittance ${{G}_{u}}$ at the pile top is defined as:

43 $$\begin{aligned} {{G}_{u}}=-\frac{2}{\theta }\sum \limits _{n=1}^{\infty }{\left\{ \frac{1+\theta [({{\eta }_{1n}}+{{\eta }_{5n}})C_{1}^{(f)}+({{\eta }_{2n}}+{{\eta }_{6n}})C_{3}^{(f)}+({{\eta }_{3n}}+{{\eta }_{7n}})C_{6}^{(f)}+({{\eta }_{4n}}+{{\eta }_{8n}})C_{7}^{(f)}]}{(E_{b}^{*}+\rho _{b}^{*}{{\delta }^{2}}{{\nu }^{2}})(h_{n}^{2}-{{\kappa }^{2}})} \right\} } \end{aligned}$$

Assuming that $s=i\omega$, and then, the dynamic stiffness ${{k}_{d}}$ and velocity amplitude-frequency response ${{H}_{v}}$ (admittance) of pile top can be obtained as:

44 $$\begin{aligned} {{k}_{d}}(i\omega )= & {} {{Z}_{u}}(i\omega ) \end{aligned}$$

45 $$\begin{aligned} \left| {{H}_{v}}(i\omega ) \right|= & {} \left| i\omega {{G}_{u}}(i\omega ) \right| \end{aligned}$$

where ${{Z}_{u}}$ denotes the complex impedance on the top of the pile segment , $\omega$ is frequency and $i=\sqrt{-1}$.

When the half-sine transient force *q*(*t*) is applied to the top of pile, the velocity response of pile top in the time domain can be derived by using convolution theorem and inverse Fourier transformation as follow:

46 $$\begin{aligned} V(t)=\frac{{{q}_{\max }}}{2\pi }\int _{-\infty }^{\infty }{\frac{2\pi {{T}_{0}}}{4{{\pi }^{2}}-{{{T}_{0}}^{2}}{{\omega }^{2}}}(1+{{e}^{{-i\omega {{T}_{0}}}/{2}\;}}){{H}_{v}}(i\omega ){{e}^{i\omega t}}d\omega } \end{aligned}$$

where ${{T}_{0}}$ represents the pulse width of the impact load.

In order to investigate the dynamic response of frozen soil layer caused by pile vibration under the transverse inertia effect, the soil impedance factor of the ${{n}^{th}}$ mode ${{\alpha }_{n}}$ can be defined as:

47 $$\begin{aligned} {{\alpha }_{n}}=\frac{{{\eta }_{1n}}C_{1}^{(f)}+{{\eta }_{2n}}C_{3}^{(f)}+{{\eta }_{3n}}C_{6}^{(f)}+{{\eta }_{4n}}C_{7}^{(f)}}{{{\eta }_{9n}}C_{1}^{(f)}+{{\eta }_{10n}}C_{3}^{(f)}+{{\eta }_{11n}}C_{6}^{(f)}+{{\eta }_{12n}}C_{7}^{(f)}} \end{aligned}$$

## Numerical results and analysis

### Validation of vertical vibration of the large diameter pile with transverse inertia effect

#### Comparison of vertical dynamic response of piles based on large-diameter pile theory considering lateral inertia effect and one-dimensional pile theory

**Figure 2:**
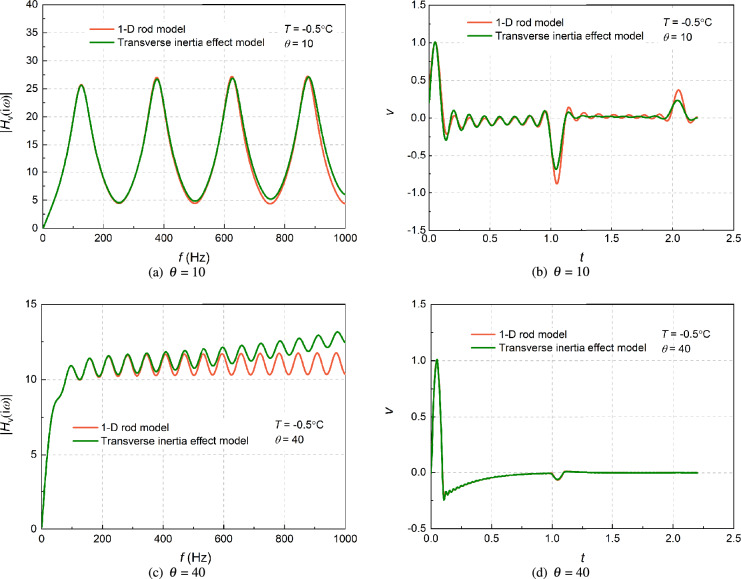
Comparisons of admittance and time-domain reflection considering the transverse inertia effect under different slenderness ratio.

In order to reflect validation of the simplified model of large-diameter pile, the admittance and reflection wave curves in the time domain of end-bearing piles with and without the transverse inertia effect under different slenderness ratios are compared. For comparisons by the numerical calculations, the pile diameter is fixed at 1.6 m, and the slenderness $\theta$ is set as 40 and 10, respectively. The temperature *T* = – 0.5°C, and the Poisson’s ratio is set as 0.30. As can be showed in Fig. 2, the dynamic responses of one-dimensional rod are basically consistent with the results of the model considering the transverse inertia effect at the low frequency. However, with the increase in frequency, the differences gradually increase, and significant differences are reflected in the time domain as well. The admittance curve shows that when the transverse inertia effect is considered, the magnitude of the admittance diverges with increasing frequency, which shows that the admittance is frequency-dependent. Moreover, the reflection curve in the time domain shows that the pile bottom reflection of the model considering the transverse inertia effect oscillates more obviously, but the peak value of the pile bottom reflection is slightly lower than that of the 1D model, indicating that the effect of dispersion is stronger under the transverse inertial effect. Furthermore, these vibration phenomena disappear gradually with the increase of $\theta$. Overall, as the slenderness ratio decreases, the differences between the two responses in the frequency domain decrease, while the differences in the time domain increase. When the $\theta$ reaches 40, the time-domain reflection curves of the two cases are basically the same, however, if the slenderness is relatively small ($\theta$ = 10), the two cases are slightly different. The result shows that if a pile is shorter and thicker, the time-domain transverse inertia effect will be more obvious.

#### Comparison of impedance factors of frozen soil layer induced by pile vibration under large-diameter and one-dimensional pile theory

**Figure 3:**
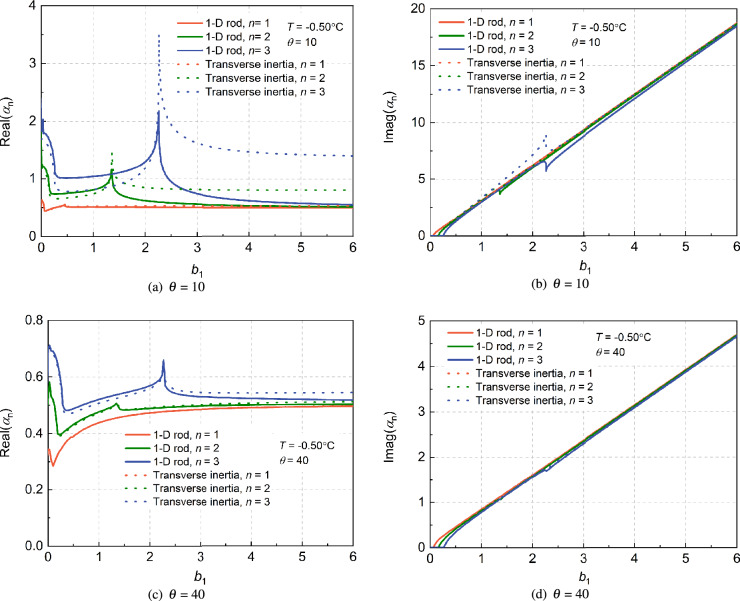
Effects of slenderness ratio on impedance factor of frozen saturated soil layer.

Figure 3 reflects the comparisons of complex impedance factors of the soil layer caused by large diameter end-bearing piles with and without transverse inertia under different slenderness. The horizontal axis in Fig. 3 is the dimensionless frequency ratio, and the vertical axis is the real or imaginary part of the impedance factors. There are n modes in the complex impedance factor of the soil layer, for simplification, the first three modes are chosen here, i.e., *n* = 1, 2, 3, respectively. It can be showed that the complex impedance factor of saturated frozen soil layer considering transverse inertia effect is basically the same as that without considering transverse inertia effect in the first mode, however, as the modal order increases, the differences in resonance frequency and amplitude increase. Li et al.^27^ has discussed in detail the reasons for the occurrence of the resonance point in saturated unfrozen soil, and established the vibration model of saturated soil layer caused by pile vibration. The impedance factor of the frozen soil layer discussed here is essentially the same as that of saturated unfrozen soil. The impedance factor of the frozen soil layer considering transverse inertia effect is significantly greater in high-order modes than in the case of not considering transverse inertia effect. Therefore, the coherence of the resonance points indicates that the vibration mode of the frozen saturated soil layer does not change when the effect of transverse inertia is taken into account, only the value of the vibration changes.

Figure 3 also reflects that the smaller the slenderness ratio ($\theta$ = 10), the larger the differences between the complex impedance factors of the frozen saturated soil layer with and without the transverse inertia effect. This indicates that, under the same pile length, the transverse inertia effect becomes more obvious as the pile diameter increases. And when the transverse inertia effect is considered, the amplitude of the complex impedance factor caused by pile vibration at the resonance point is larger than traditionally one-dimensional rod.

### Parametric analysis of the vertical vibration response of pile

#### Effect of temperature on vertically dynamic responses of large-diameter pile in frozen soil

The temperature has a significant impact on the physical and mechanical properties of frozen soil. On the one hand, it affects the unfrozen water content in frozen soil, and on the other hand, it has a significant impact on the elastic modulus and Poisson’s ratio of frozen soil. The changes in these two properties have a significant impact on the vertical vibration of piles in frozen soil.

##### Effect of temperature on unfrozen water content

The unfrozen water in frozen soil is generally divided into two parts: unfrozen water in unfrozen small pores and the water contained in unfrozen water films in freezing large pores. For convenience, it is referred to as free unfrozen water ${{\phi }_{uu}}$ and non-free unfrozen water ${{\phi }_{uf}}$. Free unfrozen water mainly depends on the pore size of the frozen soil. At a certain temperature, when it is smaller than a certain pore size, the freezing temperature decreases due to the interface curvature effect, which is related to the surface tension and pore size of ice water, so the water under this pore size needs a lower temperature to freeze. When Leclaire et al.^38^ proposed the solid-ice-liquid three-phase coupling model, they provided a formula for calculating the ice content based on free unfrozen water. They believed that pore water would not freeze at a certain temperature below a specific pore size, and therefore the unfrozen soil content was related to temperature, pore size, and surface tension. However, in comparison with the results of a large amount of unfrozen water experiments in frozen soil, it was found that it did not match well with the experimental results. The reason is that in frozen soil, except for this part of free unfrozen water, even in frozen large pores, there is still a considerable proportion of unfrozen water, known as non-free unfrozen water, due to the presence of unfrozen water films on particles and ice surfaces. It is caused by the formation of an unfrozen water film due to the surface tension of particles. In the calculation theory of unfrozen water content in frozen soil, the content of this water is generally believed to be related to capillary force, surface tension, adsorption force, and double electric layer, and is usually calculated based on the capillary force of colloids.

Wang et al. proposed a calculation method that considers these two parts of unfrozen water. For frozen particles in large pores, an unfrozen water film is formed around the soil particles due to the influence of matrix potential^44^. The thickness of the unfrozen water film can be expressed as follows:

48 $$\begin{aligned} d({{\psi }_{m}})=\root 3 \of {\frac{A}{6\pi g{{\rho }_{w}}{{\psi }_{m}}}} \end{aligned}$$

49 $$\begin{aligned} {{\psi }_{m}}=-712.38\ln (\frac{T}{{{T}_{0}}})+5.545(T-{{T}_{0}})-3.14\times {{10}^{-3}}({{T}^{2}}-T_{0}^{2})\ \end{aligned}$$

where, ${{\psi }_{m}}$ is the thickness of an absorbed thin water film, *A* is the Hamaker constant for solid-vapor interactions through the intervening liquid, and is taken as -6$\times {{10}^{-20}}$, *g* is the acceleration due to gravity, and ${{\psi }_{m}}$ is the matric head. ${{\rho }_{w}}$ denotes the average density of adsorbed thin water film, and is taken as 2000 kg/m^3^ and 1700 kg/m^3^ for solid particle and ice particle, respectively.

50 $$\begin{aligned} {{\phi }_{uf}}={{\phi }_{s}}\int _{{{T}_{0}}-\Delta {{T}_{f\min }}}^{T}{(4\pi {{r}^{2}}{{d}_{u}})/(\tfrac{4}{3}\pi {{r}^{3}})dT}\ \end{aligned}$$

The free unfrozen water content is calculated based on the pore size distribution, and referring to pore distribution function in reference^44^, the formula for free unfrozen water content is obtained as follows:

51 $$\begin{aligned} {{\phi }_{uu}}=(1-{{\phi }_{s}}){{\left\{ \frac{1}{\ln [e+{{(\frac{{{e}^{(-T+{{T}_{0}})}}}{a})}^{n}}]} \right\} }^{m}}\ \end{aligned}$$

where *a*, *m* and *n* are the experimental fitting parameters for unfrozen water in frozen soil. By adding the unfrozen water content in Eqs. (50) and (51), the total unfrozen water content in the frozen soil can be obtained as:

52 $$\begin{aligned} {{\phi }_{w}}={{\phi }_{uf}}+{{\phi }_{uu}}\ \end{aligned}$$

According to the experimental data provided in the literature on the relationship between unfrozen water content and temperature in frozen soil, it was found that the deviation of the calculation results gradually increases at lower temperatures. The main reason is the inaccurate relationship between freezing point temperature, surface tension, and pore size. A modified calculation formula for the freezing point temperature is proposed on the basis of the Gibbs Thomson effect and the average water film thickness as follows:

53 $$\begin{aligned} \ln (\frac{{{T}_{1}}}{{{T}_{0}}})=-\frac{2{{\sigma }_{iw}}}{r{{\rho }_{i}}{{L}_{w}}}\ \end{aligned}$$

where ${{T}_{0}}$ = 273.15 *K*, ${{\sigma }_{im}}$ = $3.5\times {{10}^{-2}}$ N/m, ${{\rho }_{i}}$ = 920 kg/m$^{3}$, ${{L}_{w}}$=$333.7\times {{10}^{3}}$ J/kg, and *r* is average pore diameter.

The unfrozen water content corresponding to the average water film thickness is:

54 $$\begin{aligned} {{\phi }_{uf}}={3d({{\psi }_{m}})}/{r}\;\ \end{aligned}$$

**Figure 4:**
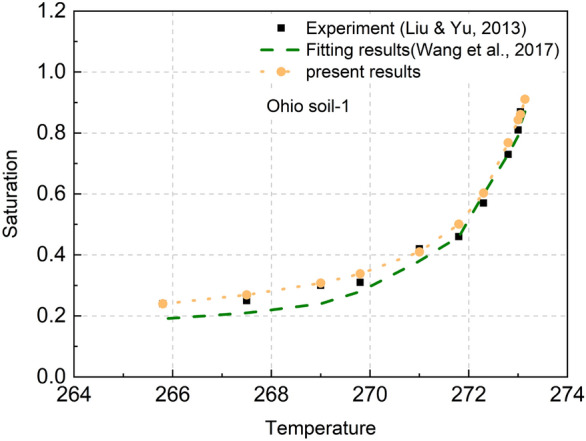
Comparison between unfrozen water content of saturated frozen soil and experimental results.

The revised calculation formula was used to obtain the unfrozen water content and saturation, and then parameter fitting was performed based on the experimental data (the parameters *a*, *m*, *n* are shown in Table 1, as shown in Fig. 4.

**Table 1:** Coefficients for saturated frozen soil and unfrozen water content.

Soil sample	Porosity	Frozen point/°C	*a*	*m*	*n*	Resource
Ohio soil-1	0.70	– 0.01	0.9706	0.5362	2.89	Liu et al.^45^
			0.98	0.438	3.60	The present method

Figure 4 shows a non-linear relationship between the unfrozen water content of saturated frozen soil and temperature. As the temperature decreases, the unfrozen water first decreases rapidly, and then the decrease rate gradually stabilizes. As shown in Fig. 4, compared with the fitting results in references^44,45^, the fitting effect of the results in the present model is better, especially at low temperatures.

##### Effect of temperature on elastic modulus and Poisson ratio of frozen soil

Furthermore, the elastic modulus and Poisson’s ratio of frozen soil are also affected by temperature, and the variation of these parameters with temperature can also affect the vertical vibration characteristics of large diameter piles. Reference^46^ summarizes a large number of experimental results. For the convenience of engineering applications, the relationship between elastic modulus, Poisson’s ratio, and temperature is summarized using a simple formula, and the following empirical calculation formula is given^47^:

55 $$\begin{aligned} {{E}_{d}}= & {} {{a}_{1}}+{{b}_{1}}{{\left| T \right| }^{m}}\ \end{aligned}$$

56 $$\begin{aligned} {{\upsilon }_{d}}= & {} {{a}_{2}}+{{b}_{2}}\left| T \right| \ \end{aligned}$$

where ${{E}_{d}}$ and ${{\upsilon }_{d}}$ denote elastic modulus and Poisson’s ratio of frozen soil, respectively. ${a}_{1}$, ${a}_{2}$, ${b}_{1}$ and ${b}_{2}$ are experimental constants, and *m* is a nonlinear exponential index, and is taken as 0.6.

##### Effect of temperature on vertical vibration of large-diameter pile in frozen soil

**Figure 5:**
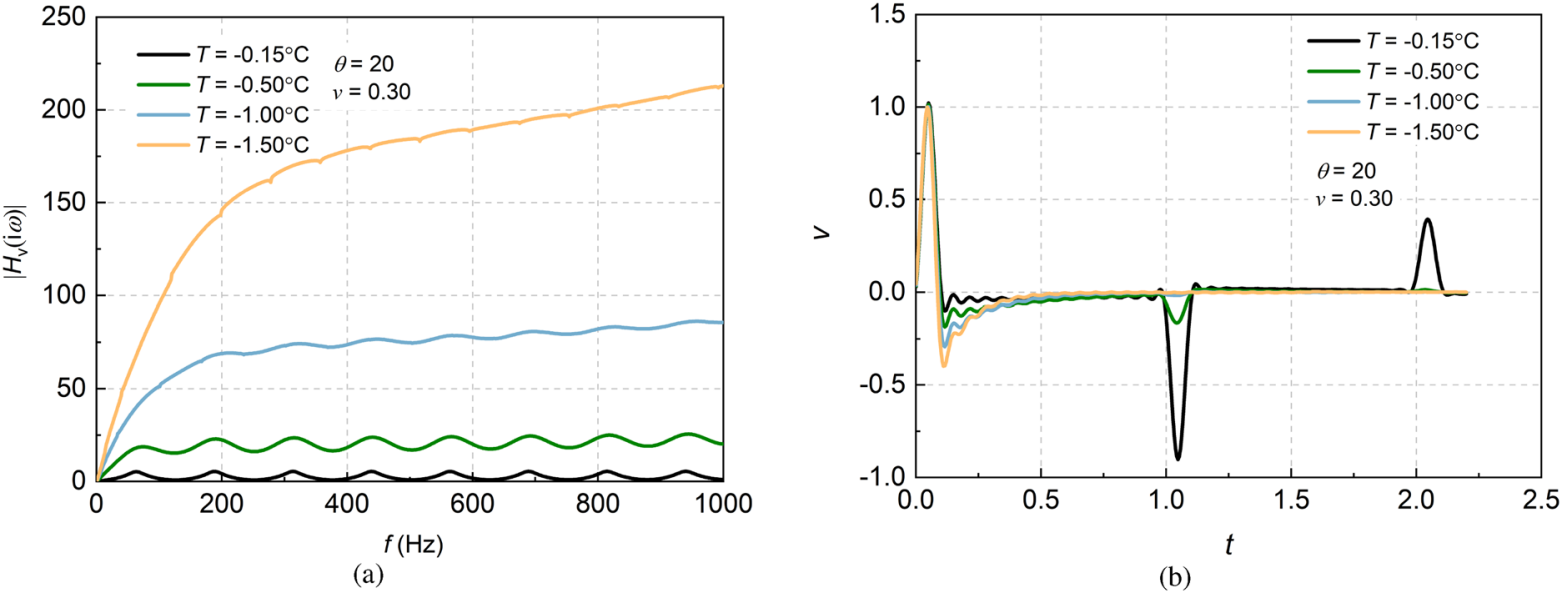
Effects of temperature on vertically dynamic responses of large-diameter pile in frozen soil.

Based on the relationship between the unfrozen water content, elastic modulus, and Poisson’s ratio with temperature, the impact on pile vertical vibration can be further investigated. The comparisons of the frequency-domain admittance and time-domain reflection of large diameter piles considering transverse inertial effect at different temperatures are shown in Fig. 5. Assuming that *T* = − 0.15 °C, − 0.50 °C, − 1.0 °C and − 1.5 °C, as seen from the Fig. 5, the admittance in the frequency domain increases sharply with the decrease in temperature, but the oscillated amplitude of admittance decreases. However, in the time-domain reflection signal, the amplitude of the reverse stretching on the incident wave increases and the reflection of the pile bottom decreases significantly. This change is due to the fact that the unfrozen water content decreases and the elastic modulus increases, consequently, the hardening of soil layer is strengthened with the decreasing temperature. Meanwhile, as the waves propagate to the bottom of the pile, the absorption of waves grows up, therefore the reflection wave signals become weaker.

#### Effect of Poisson’s ratio of pile on dynamic responses considering transverse inertia

**Figure 6:**
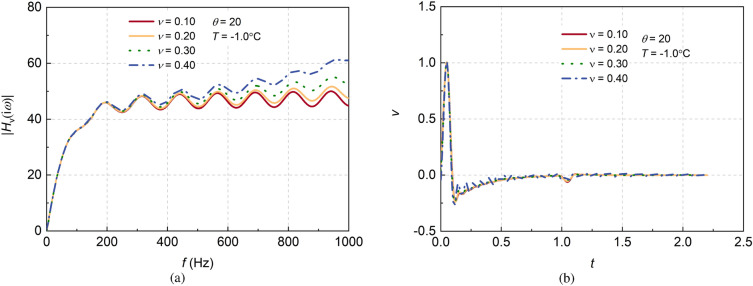
Effects of dynamic Poisson’s ratio of pile on the dynamic response considering transverse inertia effect.

Poisson’s ratio is the transverse deformation coefficient of materials. Under dynamic conditions, the dynamic Poisson’s ratio of concrete pile ranges from $\nu = 0.10\sim 0.40$. The influences of Poisson’s ratio of pile on the frequency-domain admittance and time-domain reflection of large diameter piles are shown in Fig. 6. The amplitude of admittance diverges when the transverse inertia of the pile is taken into account, and further investigation shows that the larger the Poisson’s ratio, the greater the drift of the peak admittance. Furthermore, the curves of pile integrity test in the time domain show that the reflection curves of the pile also exhibit a clear oscillation phenomenon, which increases with the increasing Poisson’s ratio.

### Parametric studies on impedance factors of frozen soil layer induced by pile vibration.

#### Effect of temperature on impedance factors of frozen soil layer

**Figure 7:**
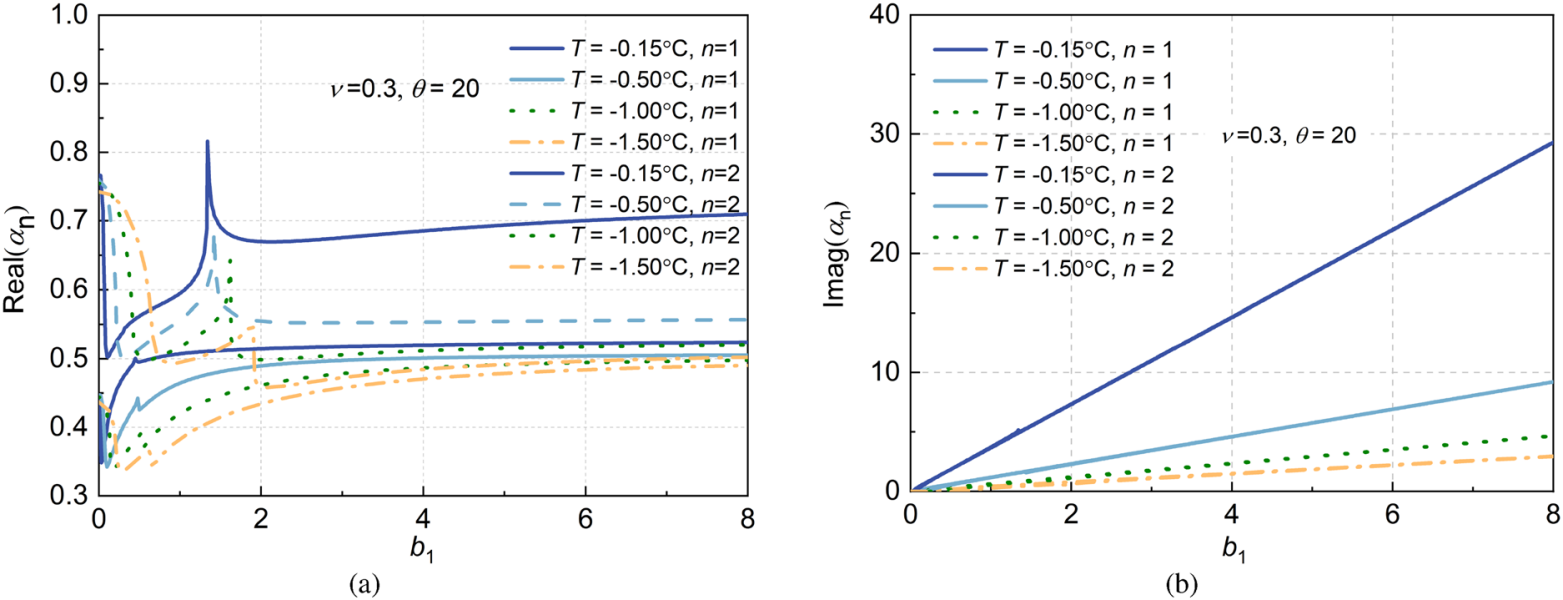
Effects of temperature on impedance factor of frozen saturated soil layer.

According to the soil impedance factor defined in Eq. (47), Fig. 7 shows the comparison of the impedance factors of the frozen soil layer of large diameter piles considering the transverse inertial effect at different temperatures. The first two orders of soil impedance factors are plotted in Fig. 7, with the temperatures set at *T*= − 0.15 °C, − 0.5 °C, − 1.0 °C and − 1.5 °C, respectively. Seen from this figure, with the decrease in temperature, the real parts of the soil layer impedance factors decrease, and the first-order soil impedance factors are significantly lower than the second-order impedance factors. While the imaginary parts of soil impedance factors linearly increase with the nondimensional frequencies, and they decrease significantly with the decrease in temperature. Furthermore, unlike the real parts, the imaginary parts of the impedance factors at the first two orders are completely consistent.

#### Effect of Poisson’s ratio of pile with transverse inertia effect

**Figure 8:**
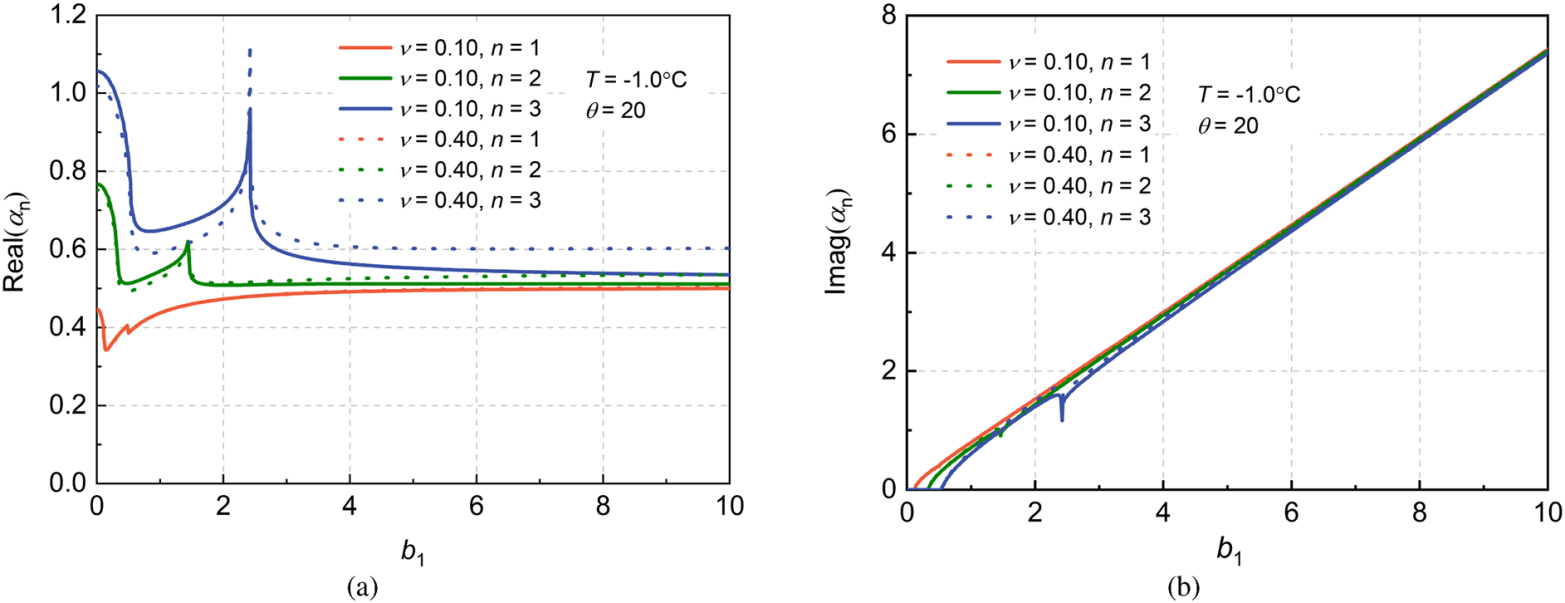
Effects of Poisson’s ratio of pile on impedance factor of frozen saturated soil.

The dynamic Poisson’s ratios of pile are chosen as $\nu =0.10$ and $\nu =0.40$ , respectively, and the influences of different Poisson’s ratios of pile on soil impedance factors are compared. It can be seen from Fig. 8 that with the increase of Poisson’s ratio, the position of the complex impedance resonance point remains unchanged. However, the magnitudes of the dynamic stiffness and dynamic damping of the frozen soil layer at the resonance point increase slightly, in particular for the high order impedance factor of the frozen soil layer.

## Conclusions

Taking into account the influence of the transverse inertia effect of pile, a theoretical model of vertical vibration of a large diameter pile in frozen saturated porous media is established. By comparing the dynamic responses of the vertical vibration of pile with the transverse inertia effect and the one-dimensional rod, and analyzing the effects of Poisson’s ratio and temperature on vertical vibration of large-diameter pile, as well as on the impedance factor of frozen soil layer, the main conclusions are listed as follow:

- When the transverse inertia effect is considered, the magnitude of the admittance diverges with increasing frequency, and the time-domain reflections of pile bottom decrease. Moreover, as the slenderness ratio decreases ($\theta$ from 40 to 10), the differences between the two responses in the frequency domain decrease, while the differences in the time domain increase.
- The complex impedance factor of saturated frozen soil layer considering transverse inertia effect is basically the same as 1-D rod model in the first mode, however, as the modal order increases (*n* = 3), the differences in resonance frequency and the amplitude increases by approximately 1.75 times compared to the 1-D model.
- The temperature has a significant impact on the unfrozen water content, the elastic modulus and Poisson’s ratio of the frozen soil. The unfrozen water contents of frozen soil proposed by the present paper are in good agreement with the experimental results, especially at low temperatures.
- With the decrease in temperature (*T* = − 0.15 °C ~ − 1.50 °C), the admittance in the frequency domain increases about 5, 15, and 40 times, respectively, and the reflection of the pile bottom gradually decreases and eventually disappear at the temperature of − 1.50 °C. Furthermore, the real parts of the soil layer impedance factors decrease, and the first-order soil impedance factors are significantly lower than the second-order impedance factors, but the imaginary parts of the impedance factors at the first two orders are completely consistent.
- With the increase in Poisson’s ratio of pile ($\nu = 0.10\sim 0.40$), the drift of the peak admittance at high frequency (after 325 Hz) increases, and the reflection curves of the pile in the time domain also exhibit a clear oscillation phenomenon. Moreover, the magnitudes of the dynamic stiffness and dynamic damping of the higher order impedance factor is obviously affected by the increasing Poisson’s ratio.

### Supplementary Information

Supplementary Information.

## Data Availability

The datasets used during the current study are available from the corresponding author upon reasonable request.
