# Supplementary material for: Dynamic response of a large-diameter end-bearing pile in permafrost
Source: Sci Rep. 2024 Jan 5;14:582. doi: 10.1038/s41598-023-46639-2 (PMC10770332; doi:10.1038/s41598-023-46639-2)
Supplement: Supplementary file 1 — Supplementary Information. [file 41598_2023_46639_MOESM1_ESM.docx]

# Dynamic response of a large-diameter end-bearing pile in permafrost

Qiang Li^1,*^, Yongyuan Zhang^1^, Chen Chen^2^, Minjie Wen^3^, Wenjie Guan^1^ and Weiwei Duan^1^

^1^Department of Civil Engineering, Zhejiang Ocean University, Zhoushan 316022, China
^2^School of Mechanics and Engineering Science, Shanghai University, Shanghai 200444, China

^3^School of Civil Engineering and Architecture, Zhejiang Sci-Tech University, Hangzhou 310018, China
^*^corresponding author email: qiangli@zjou.edu.cn

**Supplementary Equations**

For axisymmetric conditions:

$$\begin{matrix} \tilde{\sigma}_{z}^{(s)}=2[\frac{\partial\tilde{u}_{z}^{(s)}}{\partial z}-\frac{1}{3}(\frac{\partial\tilde{u}_{r}^{(s)}}{\partial r}+\frac{\tilde{u}_{r}^{(s)}}{r}+\frac{\partial\tilde{u}_{z}^{(s)}}{\partial z})]+K_{1}^{*}(\frac{\partial\tilde{u}_{r}^{(s)}}{\partial r}+\frac{\tilde{u}_{r}^{(s)}}{r}+\frac{\partial\tilde{u}_{z}^{(s)}}{\partial z}) \\ +C_{12}^{*}(\frac{\partial\tilde{u}_{r}^{(f)}}{\partial r}+\frac{\tilde{u}_{r}^{(f)}}{r}+\frac{\partial\tilde{u}_{z}^{(f)}}{\partial z}) \\ =2\frac{\partial\tilde{u}_{z}^{(s)}}{\partial z}+(K_{1}^{*}-\frac{2}{3}\mu_{11}^{*})(\frac{\partial\tilde{u}_{r}^{(s)}}{\partial r}+\frac{\tilde{u}_{r}^{(s)}}{r}+\frac{\partial\tilde{u}_{z}^{(s)}}{\partial z})+C_{12}^{*}(\frac{\partial\tilde{u}_{r}^{(f)}}{\partial r}+\frac{\tilde{u}_{r}^{(f)}}{r}+\frac{\partial\tilde{u}_{z}^{(f)}}{\partial z}) \end{matrix}$$

$\begin{matrix} \tilde{\tau}_{zr}^{(s)}=2(\varepsilon_{zr}^{(s)}-\varepsilon_{m}^{(s)}\delta_{zr})+(K_{1}^{*}\theta_{1}+C_{12}^{*}\theta_{2})\delta_{zr} \\ =(\frac{\partial\tilde{u}_{z}^{(s)}}{\partial r}+\frac{\partial\tilde{u}_{r}^{(s)}}{\partial z}) \end{matrix}$ $\tilde{\sigma}^{(f)}=C_{12}^{*}(\frac{\partial\tilde{u}_{r}^{(s)}}{\partial r}+\frac{\tilde{u}_{r}^{(s)}}{r}+\frac{\partial\tilde{u}_{z}^{(s)}}{\partial z})+K_{2}^{*}(\frac{\partial\tilde{u}_{r}^{(f)}}{\partial r}+\frac{\tilde{u}_{r}^{(f)}}{r}+\frac{\partial\tilde{u}_{z}^{(f)}}{\partial z})+C_{23}^{*}(\frac{\partial\tilde{u}_{r}^{(i)}}{\partial r}+\frac{\tilde{u}_{r}^{(i)}}{r}+\frac{\partial\tilde{u}_{z}^{(i)}}{\partial z})$

$$\tilde{\sigma}_{z}^{(i)}=2\mu_{33}^{*}\frac{\partial\tilde{u}_{z}^{(i)}}{\partial z}+(K_{3}^{*}-\frac{2}{3})(\frac{\partial\tilde{u}_{r}^{(i)}}{\partial r}+\frac{\tilde{u}_{r}^{(i)}}{r}+\frac{\partial\tilde{u}_{z}^{(i)}}{\partial z})+C_{23}^{*}(\frac{\partial\tilde{u}_{r}^{(f)}}{\partial r}+\frac{\tilde{u}_{r}^{(f)}}{r}+\frac{\partial\tilde{u}_{z}^{(f)}}{\partial z})$$

$$\begin{matrix} \tilde{\tau}_{zr}^{(i)}=2\mu_{33}^{*}(\varepsilon_{zr}^{(i)}-\varepsilon_{m}^{(i)}\delta_{zr})+(K_{3}^{*}\theta_{3}+C_{23}^{*}\theta_{2})\delta_{zr} \\ =\mu_{33}^{*}(\frac{\partial\tilde{u}_{z}^{(i)}}{\partial r}+\frac{\partial\tilde{u}_{r}^{(i)}}{\partial z}) \end{matrix}$$

Substituting in the previous expression, we get:

$$\begin{matrix} \tilde{\sigma}_{z}^{(s)}=\left\{[\bar{\lambda}_{1}(g_{11}^{2}+g_{12}^{2})+2g_{12}^{2}]C_{1}^{(s)}+C_{12}^{*}(g_{11}^{2}+g_{12}^{2})C_{1}^{(f)}\right\}k_{0}(g_{11}r)e^{g_{12}\tilde{z}} \\ +\left\{[\bar{\lambda}_{1}(g_{11}^{2}+g_{12}^{2})+2g_{12}^{2}]D_{1}^{(s)}+C_{12}^{*}(g_{11}^{2}+g_{12}^{2})D_{1}^{(f)}\right\}k_{0}(g_{11}\tilde{r})e^{-g_{12}\tilde{z}} \\ +\left\{[\bar{\lambda}_{1}(g_{21}^{2}+g_{22}^{2})+2g_{22}^{2}]C_{2}^{(s)}+C_{12}^{*}(g_{21}^{2}+g_{22}^{2})C_{2}^{(f)}\right\}k_{0}(g_{21}\tilde{r})e^{g_{22}\tilde{z}} \\ +\left\{[\bar{\lambda}_{1}(g_{21}^{2}+g_{22}^{2})+2g_{22}^{2}]D_{2}^{(s)}+C_{12}^{*}(g_{21}^{2}+g_{22}^{2})D_{2}^{(f)}\right\}k_{0}(g_{21}\tilde{r})e^{-g_{22}\tilde{z}} \\ +\left\{[\bar{\lambda}_{1}(g_{31}^{2}+g_{32}^{2})+2g_{32}^{2}]C_{3}^{(s)}+C_{12}^{*}(g_{31}^{2}+g_{32}^{2})C_{3}^{(f)}\right\}k_{0}(g_{31}\tilde{r})e^{g_{32}\tilde{z}} \\ +\left\{[\bar{\lambda}_{1}(g_{31}^{2}+g_{32}^{2})+2g_{32}^{2}]D_{2}^{(s)}+C_{12}^{*}(g_{31}^{2}+g_{32}^{2})D_{2}^{(f)}\right\}k_{0}(g_{31}\tilde{r})e^{-g_{32}\tilde{z}} \\ -2g_{41}^{2}g_{42}C_{6}^{(s)}k_{0}(g_{41}\tilde{r})e^{g_{42}\tilde{z}}+2g_{41}^{2}g_{42}D_{6}^{(s)}k_{0}(g_{41}\tilde{r})e^{-g_{42}\tilde{z}} \\ -2g_{51}^{2}g_{52}C_{7}^{(s)}k_{0}(g_{51}\tilde{r})e^{g_{52}\tilde{z}}+2g_{51}^{2}g_{52}D_{7}^{(s)}k_{0}(g_{51}\tilde{r})e^{-g_{52}\tilde{z}} \end{matrix}$$

$$\begin{matrix} \tilde{\sigma}^{(f)}=\left\{C_{12}^{*}(g_{11}^{2}+g_{12}^{2})C_{1}^{(s)}+K_{2}^{*}(g_{11}^{2}+g_{12}^{2})C_{1}^{(f)}+C_{23}^{*}(g_{11}^{2}+g_{12}^{2})C_{1}^{(i)}\right\}k_{0}(g_{11}\tilde{r})e^{g_{12}\tilde{z}} \\ +\left\{C_{12}^{*}(g_{11}^{2}+g_{12}^{2})D_{1}^{(s)}+K_{2}^{*}(g_{11}^{2}+g_{12}^{2})D_{1}^{(f)}+C_{23}^{*}(g_{11}^{2}+g_{12}^{2})D_{1}^{(i)}\right\}k_{0}(g_{11}\tilde{r})e^{-g_{12}\tilde{z}} \\ +\left\{C_{12}^{*}(g_{21}^{2}+g_{22}^{2})C_{2}^{(s)}+K_{2}^{*}(g_{21}^{2}+g_{22}^{2})C_{2}^{(f)}+C_{23}^{*}(g_{21}^{2}+g_{22}^{2})C_{2}^{(i)}\right\}k_{0}(g_{21}\tilde{r})e^{g_{22}\tilde{z}} \\ +\left\{C_{12}^{*}(g_{21}^{2}+g_{22}^{2})D_{2}^{(s)}+K_{2}^{*}(g_{21}^{2}+g_{22}^{2})D_{2}^{(f)}+C_{23}^{*}(g_{21}^{2}+g_{22}^{2})D_{2}^{(i)}\right\}k_{0}(g_{21}\tilde{r})e^{-g_{22}\tilde{z}} \\ +\left\{C_{12}^{*}(g_{31}^{2}+g_{32}^{2})C_{3}^{(s)}+K_{2}^{*}(g_{31}^{2}+g_{32}^{2})C_{3}^{(f)}+C_{23}^{*}(g_{31}^{2}+g_{32}^{2})C_{3}^{(i)}\right\}k_{0}(g_{31}\tilde{r})e^{g_{32}\tilde{z}} \\ +\left\{C_{12}^{*}(g_{31}^{2}+g_{32}^{2})D_{3}^{(s)}+K_{2}^{*}(g_{31}^{2}+g_{32}^{2})D_{3}^{(f)}+C_{23}^{*}(g_{31}^{2}+g_{32}^{2})D_{3}^{(i)}\right\}k_{0}(g_{31}\tilde{r})e^{-g_{32}\tilde{z}} \end{matrix}$$

$$\begin{matrix} \tilde{\sigma}_{z}^{(i)}=\left\{[\bar{\lambda}_{3}(g_{11}^{2}+g_{12}^{2})+2\mu_{3}^{*}g_{12}^{2}]C_{1}^{(i)}+C_{23}^{*}(g_{11}^{2}+g_{12}^{2})C_{1}^{(f)}\right\}k_{0}(g_{11}\tilde{r})e^{g_{12}\tilde{z}} \\ +\left\{[\bar{\lambda}_{3}(g_{11}^{2}+g_{12}^{2})+2\mu_{3}^{*}g_{12}^{2}]D_{1}^{(i)}+C_{23}^{*}(g_{11}^{2}+g_{12}^{2})D_{1}^{(f)}\right\}k_{0}(g_{11}\tilde{r})e^{-g_{12}\tilde{z}} \\ +\left\{[\bar{\lambda}_{3}(g_{21}^{2}+g_{22}^{2})+2\mu_{3}^{*}g_{22}^{2}]C_{2}^{(i)}+C_{23}^{*}(g_{21}^{2}+g_{22}^{2})C_{2}^{(f)}\right\}k_{0}(g_{21}\tilde{r})e^{g_{22}\tilde{z}} \\ +\left\{[\bar{\lambda}_{3}(g_{21}^{2}+g_{22}^{2})+2\mu_{3}^{*}g_{22}^{2}]D_{2}^{(i)}+C_{23}^{*}(g_{21}^{2}+g_{22}^{2})D_{2}^{(f)}\right\}k_{0}(g_{21}\tilde{r})e^{-g_{22}\tilde{z}} \\ +\left\{[\bar{\lambda}_{3}(g_{31}^{2}+g_{32}^{2})+2\mu_{3}^{*}g_{32}^{2}]C_{3}^{(i)}+C_{23}^{*}(g_{31}^{2}+g_{32}^{2})C_{3}^{(f)}\right\}k_{0}(g_{31}\tilde{r})e^{g_{32}\tilde{z}} \\ +\left\{[\bar{\lambda}_{3}(g_{31}^{2}+g_{32}^{2})+2\mu_{3}^{*}g_{32}^{2}]D_{3}^{(i)}+C_{23}^{*}(g_{31}^{2}+g_{32}^{2})D_{3}^{(f)}\right\}k_{0}(g_{31}\tilde{r})e^{-g_{32}\tilde{z}} \\ -2\mu_{3}^{*}g_{41}^{2}g_{42}C_{6}^{(i)}k_{0}(g_{41}\tilde{r})e^{g_{42}\tilde{z}}+2\mu_{3}^{*}g_{41}^{2}g_{42}D_{6}^{(i)}k_{0}(g_{41}\tilde{r})e^{-g_{42}\tilde{z}} \\ -2\mu_{3}^{*}g_{51}^{2}g_{52}C_{7}^{(i)}k_{0}(g_{51}\tilde{r})e^{g_{52}\tilde{z}}+2\mu_{3}^{*}g_{51}^{2}g_{52}D_{7}^{(i)}k_{0}(g_{51}\tilde{r})e^{-g_{52}\tilde{z}} \end{matrix}$$

$$\begin{matrix} \tilde{\tau}_{rz}^{(s)}=-2g_{11}g_{12}C_{1}^{(s)}k_{1}(g_{11}\tilde{r})e^{g_{12}\tilde{z}}+2g_{11}g_{12}D_{1}^{(s)}k_{1}(g_{11}\tilde{r})e^{-g_{12}\tilde{z}} \\ -2g_{21}g_{22}C_{2}^{(s)}k_{1}(g_{21}\tilde{r})e^{g_{22}\tilde{z}}+2g_{21}g_{22}D_{2}^{(s)}k_{1}(g_{21}\tilde{r})e^{-g_{22}\tilde{z}} \\ -2g_{31}g_{32}C_{3}^{(s)}k_{1}(g_{31}\tilde{r})e^{g_{32}\tilde{z}}+2g_{31}g_{32}D_{3}^{(s)}k_{1}(g_{31}\tilde{r})e^{-g_{32}\tilde{z}} \\ +(g_{41}^{3}-g_{41}g_{42}^{2})C_{6}^{(s)}k_{1}(g_{41}\tilde{r})e^{g_{42}\tilde{z}}+(g_{41}^{3}-g_{41}g_{42}^{2})D_{6}^{(s)}k_{1}(g_{41}\tilde{r})e^{-g_{42}\tilde{z}} \\ +(g_{51}^{3}-g_{51}g_{52}^{2})C_{7}^{(s)}k_{1}(g_{51}\tilde{r})e^{g_{52}\tilde{z}}+(g_{51}^{3}-g_{51}g_{52}^{2})D_{7}^{(s)}k_{1}(g_{51}\tilde{r})e^{-g_{52}\tilde{z}} \end{matrix}$$

$$\begin{matrix} \tilde{\tau}_{rz}^{(i)}=-2\mu_{3}^{*}g_{11}g_{12}C_{1}^{(i)}k_{1}(g_{11}\tilde{r})e^{g_{12}\tilde{z}}+2\mu_{3}^{*}g_{11}g_{12}D_{1}^{(i)}k_{1}(g_{11}\tilde{r})e^{-g_{12}\tilde{z}} \\ -2\mu_{3}^{*}g_{21}g_{22}C_{2}^{(i)}k_{1}(g_{21}\tilde{r})e^{g_{22}\tilde{z}}+2\mu_{3}^{*}g_{21}g_{22}D_{2}^{(i)}k_{1}(g_{21}\tilde{r})e^{-g_{22}\tilde{z}} \\ -2\mu_{3}^{*}g_{31}g_{32}C_{3}^{(i)}k_{1}(g_{31}\tilde{r})e^{g_{32}\tilde{z}}+2\mu_{3}^{*}g_{31}g_{32}D_{3}^{(i)}k_{1}(g_{31}\tilde{r})e^{-g_{32}\tilde{z}} \\ +\mu_{3}^{*}(g_{41}^{3}-g_{41}g_{42}^{2})C_{6}^{(i)}k_{1}(g_{41}\tilde{r})e^{g_{42}\tilde{z}}+\mu_{3}^{*}(g_{41}^{3}-g_{41}g_{42}^{2})D_{6}^{(i)}k_{1}(g_{41}\tilde{r})e^{-g_{42}\tilde{z}} \\ +\mu_{3}^{*}(g_{51}^{3}-g_{51}g_{52}^{2})C_{7}^{(i)}k_{1}(g_{51}\tilde{r})e^{g_{52}z}+\mu_{3}^{*}(g_{51}^{3}-g_{51}g_{52}^{2})D_{7}^{(i)}k_{1}(g_{51}\tilde{r})e^{-g_{52}\tilde{z}} \end{matrix}$$
